# An Advanced Systems Pharmacology Strategy Reveals *AKR1B1, MMP2, PTGER3* as Key Genes in the Competing Endogenous RNA Network of Compound Kushen Injection Treating Gastric Carcinoma by Integrated Bioinformatics and Experimental Verification

**DOI:** 10.3389/fcell.2021.742421

**Published:** 2021-09-27

**Authors:** Wei Zhou, Chao Wu, Chongjun Zhao, Zhihong Huang, Shan Lu, Xiaotian Fan, Yingying Tan, Antony Stalin, Rongli You, Xinkui Liu, Jingyuan Zhang, Zhishan Wu, Jiarui Wu

**Affiliations:** ^1^Department of Clinical Chinese Pharmacy, School of Chinese Materia Medica, Beijing University of Chinese Medicine, Beijing, China; ^2^China-Japan Friendship Hospital, Beijing, China; ^3^Beijing Key Laboratory for Quality Evaluation of Chinese Materia Medica, School of Chinese Materia Medica, Beijing University of Chinese Medicine, Beijing, China; ^4^State Key Laboratory of Subtropical Silviculture, Department of Traditional Chinese Medicine, Zhejiang A&F University, Hangzhou, China; ^5^Shanxi Zhendong Pharmaceutical Co., Ltd., Shanxi, China

**Keywords:** compound Kushen injection, gastric cancer, ceRNA network, system pharmacology, meta-analysis

## Abstract

Gastric carcinoma (GC) is a severe tumor of the digestive tract with high morbidity and mortality and poor prognosis, for which novel treatment options are urgently needed. Compound Kushen injection (CKI), a classical injection of Chinese medicine, has been widely used to treat various tumors in clinical practice for decades. In recent years, a growing number of studies have confirmed that CKI has a beneficial therapeutic effect on GC, However, there are few reports on the potential molecular mechanism of action. Here, using systems pharmacology combined with proteomics analysis as a core concept, we identified the ceRNA network, key targets and signaling pathways regulated by CKI in the treatment of GC. To further explore the role of these key targets in the development of GC, we performed a meta-analysis to compare the expression differences between GC and normal gastric mucosa tissues. Functional enrichment analysis was further used to understand the biological pathways significantly regulated by the key genes. In addition, we determined the significance of the key genes in the prognosis of GC by survival analysis and immune infiltration analysis. Finally, molecular docking simulation was performed to verify the combination of CKI components and key targets. The anti-gastric cancer effect of CKI and its key targets was verified by *in vivo* and *in vitro* experiments. The analysis of ceRNA network of CKI on GC revealed that the potential molecular mechanism of CKI can regulate PI3K/AKT and Toll-like receptor signaling pathways by interfering with hub genes such as *AKR1B1, MMP2* and *PTGERR3*. In conclusion, this study not only partially highlighted the molecular mechanism of CKI in GC therapy but also provided a novel and advanced systems pharmacology strategy to explore the mechanisms of traditional Chinese medicine formulations.

## Introduction

Gastric carcinoma (GC) is a malignant tumor of the digestive system that poses a serious threat to human health (Ajani et al., 2017). According to the statistics of International Agency for Research on Cancer, there were about 1.08 million new cases of gastric cancer worldwide in 2020, and about 0.77 million of people die from gastric cancer (Bray et al., 2018; Sung et al., 2021). The morbidity and mortality of GC have declined sharply in recent decades in some Western countries, while it is still relatively high in East Asia and represents a significant medical burden (Ferro et al., 2014; Torre et al., 2015). Among the factors that increase the risk of human GC, *Helicobacter pylori* gastric infection plays a vital role, and 75% of GC cases worldwide are caused by *Helicobacter pylori* infection (Plummer et al., 2015). Although early stage GC is highly treatable, the median survival time of advanced GC is only 9–10 months. Unsatisfactorily, the global 5-year survival rate of patients is still less than 30% (Verdecchia et al., 2007). The combination of different forms and different drugs of chemotherapy, radiotherapy and surgery are common treatment methods to treat GC (Coccolini, 2016). However, because of the internal metastasis and changes of the tumor, the heterogeneity of different patients and the side effects of radiotherapy and chemotherapy, patients’ options in clinical practice are minimal (Ajani et al., 2017).

Compound Kushen injection (CKI), also called Yanshu injection, consists of Kushen (Radix Sophorae flavescentis) and Baituling (Rhizoma Smilacisglabrae) (Zhao et al., 2014). CKI has been adopted clinically for a decade to treat various solid tumors, including GC, liver cancer, lung cancer, breast cancer and other cancers (Xu et al., 2011; Sun et al., 2012; Wang et al., 2015). It is worth mentioning that CKI can also relieve cancer pain, regulate immunity, and improve conventional chemotherapy to reduce tumor efficacy and chemotherapy toxicity (Tu et al., 2016; Yang et al., 2020). The anti-tumor effect of CKI has been confirmed while the underlying molecular mechanism is still poorly understood.

Molecular studies have provided a vast quantity of new information for potential mechanisms to use in cancer treatment. Microarray and high-throughput sequencing technologies provide a reliable guarantee for the deciphering of significant genetic or epigenetic alterations in carcinogenesis and the discovery of potential biomarkers for cancer diagnosis, treatment, and prognosis (Cancer Genome Atlas Research Network, 2014). MicroRNA (miRNA) and long noncoding RNA (lncRNA) are the two most common subtypes of noncoding RNA (ncRNA). Their abnormality leads to the inability of mRNA to be transcribed normally and may contribute to unhindered growth, and invasion of cancer cells (Schmitt and Chang, 2016; Rupaimoole and Slack, 2017). At present, studies have confirmed that the lncRNA-miRNA-mRNA network plays a vital role in the occurrence and development of cancer, which may have substantial clinical prospects for identifying potential biomarkers and therapeutic targets of various tumors (Liu et al., 2019, 2020).

**FIGURE 1:**
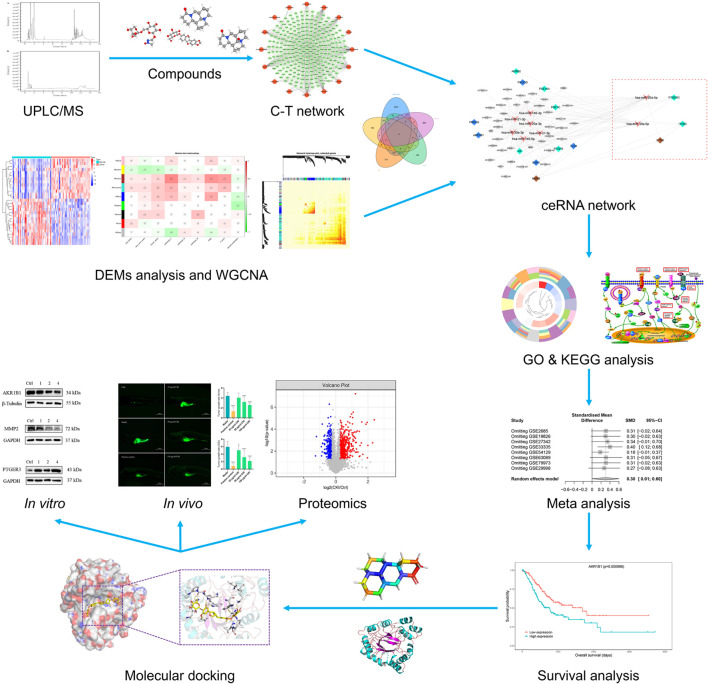
The workflow of the advanced systems pharmacology strategy.

Thus, in this study, we firstly analyzed the microarray dataset in the Gene Expression Omnibus (GEO) database and The Cancer Genome Atlas (TCGA) to find miRNAs that are differentially expressed in GC compared to normal tissues. Secondly, we applied weighted gene co-expression network analysis (WGCNA) and merged with differentially expressed miRNAs (DEMs) predicted targets to identify genes associated with GC progression systematically. Afterward, we undertake a systematic study of the molecular mechanism of CKI in the treatment of GC using a network pharmacology analytical model and proteomics analysis. Drawing on the above research, we conducted a meta-analysis of key targets to verify their expression changes in GC and conduct immune infiltration to explore their prognostic impact on GC patients. At last, to better analyze and predict the molecular mechanism of CKI on GC, enrichment analysis, molecular docking and biological experiments were exploited to discover and verify the involved pathways and the binding of CKI components to key targets. Figure 1 depicts a workflow of the advanced systems pharmacology strategy used in this study.

## Materials and Methods

### Detection of Ingredients in Compound Kushen Injection

Compound Kushen injection (batch number: 20200329) was supplied by Zhendong Pharmaceutical Co., Ltd. (Shanxi, China). UPLC-QE-Orbitrap-MS Separation was performed on a Waters HSS T3 UPLC C18 analytical column (1.7 μm, 2.1 mm × 100 mm, Milford, MA, United States). The oven was set at 40°C; the injection volume was 5 μL; the flow rate was set at 0.3 mL/min; the mobile phase was consisted of 0.1% formic acid in water (A) and carbinol (B). The eluting program was: 5–5% B for 0–1 min, 5–95%B for 1–12 min, 95–95%B for 12–15 min. The ion source was electrospray ionization (ESI); MS was operated in positive mode; the scan mode was Full scan/ddMS2, the scan range was 100–1200 Da; The capillary temperature was 350°C; The spray voltage in negative mode was 3800 V; The spray voltage in positive mode was 3200 V; The sheath gas was 35 arb; The aux gas was 15 arb; three collision energies of low, medium and high were used for MS2. The positive ion mode was 30, 40, 50 eV, and the negative ion mode was 30, 50, 70 eV. The resolution of the full scan was 70000 FWHM, and the resolution of MS2 was 17500 FWHM. The reference marker compounds present in the sample were identified based on retention time, MS fragmentation and UV spectra.

### Construction of Compound Kushen Injection Ingredient Prediction Target Network

For a more profound and comprehensive study, we input the 3D structure of chemical composition in Traditional Chinese Medicine Systems Pharmacology Database and Analysis Platform (TCMSP) (Ru et al., 2014), Search Tool for Interactions of Chemicals (STITCH) (Szklarczyk et al., 2016), SuperPred (Nickel et al., 2014), SwissTargetPrediction (Gfeller et al., 2014) for target prediction. Moreover, the predicted multiple target information of the compounds and the obtained information was introduced into Cytoscape 3.6.1 (Franz et al., 2016)^1^ to obtain an intermolecular interaction network and carry out complex network analyses.

### Differentially Expressed MicroRNA Analysis and Target Prediction of Gastric Carcinoma

#### Differential Expression of MicroRNAs in Gastric Carcinoma

Microarray data for gene expression GSE23739 were downloaded from the GEO database. A total of 80 samples were obtained, including 40 primary tumors and 40 normal samples. After the raw data has undergone background correction and standardization, the Limma (Ritchie et al., 2015) R package was applied to analyze the difference between cancer and normal tissues. The miRNA-seq data in TCGA contains 446 tumor samples and 45 normal samples. To verify and obtain DEMs, the edgeR (Robinson et al., 2010) package was used to analyze the difference between the groups.

#### Differentially Expressed MicroRNAs Target Genes Prediction

MiRWalk2.0 (Dweep and Gretz, 2015) is a comprehensive archive that fully integrates the interactions of multiple existing miRNA target prediction databases and provides predictive and experimentally verified miRNA target prediction. On the one hand, the interactions between miRNAs and genes from 12 servers were speculated and only the genes predicted by more than six of the servers were identified as target genes. On the other hand, 5 servers using miRWalk, miRanda, PITA, RNAhybrid and Targetscan were utilized to predict miRNA-lncRNA targets.

### Weighted Gene Co-expression Network Analysis for Gastric Carcinoma mRNA

#### Data Collection and Preprocessing

The TCGA-STAD RNA-seq data includes 407 samples of its HTSeq-Counts data and associated clinical information downloaded in February 2020. After removing samples that contained incomplete analytical data and/or other malignancies, 375 samples were retained. Because, some genes without significant changes in expression between samples, we selected the top 5000 genes that are most relevant to differential expression for the following WGCNA analysis.

#### Weighted Gene Co-expression Network Analysis and Module Preservation

The gene co-expression networks were constructed using the WGCNA package (Langfelder and Horvath, 2008). The similarity between gene expression profiles was used to construct a similarity matrix based on pairwise Pearson correlation coefficient matrices. To improve co-expression similarity and achieve a scale-free topology, an appropriate soft threshold power β was selected by using the integration function (pickSoftThresshold) in the WGCNA software package (Jeong et al., 2001). We also reconstructed the topological overlap matrix by calculating the Topological Overlap Measure (TOM) which is a robust measure of network interconnectedness (Li and Horvath, 2007; Yip and Horvath, 2007). Finally, the Dynamic Tree-Cut algorithm method was applied to identify the module of gene co-expression with maxBlockSize of 6000, minModuleSize of 30 and mergeCutHeight of 0.2.

#### Identification of Clinical Significant Modules

The module eigengene (ME) is the first principal component of each gene module and the expression of ME is considered representative of all genes in one module. The Module Membership (MM) is the correlation between the ME and the gene expression profile. Gene Significance (GS) is the absolute value of the correlation between a specific gene and a clinical trait. According to ME, GS, MM, we can associate modules with clinical traits, not only to calculate the correlation between ME and clinical traits, but also to analyze clinically vital modules (Langfelder and Horvath, 2008).

### Prediction of Competing Endogenous RNA Network of Compound Kushen Injection Intervention in Gastric Carcinoma

To systematically describe GC-associated underlying molecular mechanism, a competing endogenous RNA (ceRNA) network was conducted by merging the predictive correlation of DEMs and key modules in WGCNA. The target network predicted for CKI active component was combined with the ceRNA network of GC for CKI intervention in GC ceRNA network prediction, and the overlapping proteins in the two networks are likely to be the potential key gene for GC treatment by CKI active ingredients. Cytoscape constructed a potential ceRNA network for CKI treatment of GC, and the potential targets for CKI treatment of gastric cancer were systematically analyzed.

### Functional Enrichment Analysis

To analyze the enrichment of key proteins, we first used the Search Tool for the Retrieval of Interacting Genes/Proteins (STRING) 10.5^2^ database to construct a protein-protein interaction (PPI) network for key proteins (Szklarczyk et al., 2017). The STRING database aims to collect, score, and integrate all publicly available sources of protein–protein interaction information, and to complement these with computational predictions. Its goal is to achieve a comprehensive and objective global network, including direct (physical) as well as indirect (functional) -interactions. We performed the Gene Ontology (GO) Functional and Kyoto Encyclopedia of Genes and Genomes (KEGG) pathway enrichment analysis for the predicted key targets of CKI compounds used in GC therapy to identify their biological functions. In addition, the R package clusterProfiler was used to perform GO and KEGG functional enrichment analysis. Particularly, the function and pathway enrichment analyses of the validated target genes of miRNAs, were used by the DIANA tool, which is based on the collaboration of the previously mentioned database (TarBase v7.0) and mirPathv3.0 (a miRNA pathway analysis web server deciphering miRNA function with experimental support) (Paraskevopoulou et al., 2016; Vlachos and Hatzigeorgiou, 2017).

### Comprehensive Meta-Analysis of the Hub Gene

#### Data Collection

A microarray search for hub genes was conducted in the GEO database with the following terms: (“stomach neoplasms”[MeSH Terms] OR gastric cancer[All Fields]) AND “Homo sapiens”[porgn] AND (“gse”[Filter] AND “Expression profiling by array”[Filter]) and the latest searching time was April 5, 2020. The criteria for inclusion were as follows: (1) patients diagnosed with gastric cancer were investigated; (2) cancerous and noncancerous samples were involved; (3) datasets samples were no less than 20. In addition, the following conditions caused the exclusion of a study: (1) lack of original data; (2) the patients with gastric cancer were accompanied by other tumors (3) the interventions included surgery, radiotherapy or other cancer treatments.

#### Statistical Analysis and Comprehensive Meta-Analysis

The expression profiling information of the datasets were exploited to calculate the mean (M) and standard deviation (SD) for each hub gene in the experimental control group. Then, the meta package of R software was brought into play the standardized meta-difference (SMD) and 95% confidential interval (CI) analysis. In addition to determining a reasonable choice of random effects and fixed effects models and evaluate heterogeneity, the chi-squared test of Q and the I2 statistic were calculated.

### Survival Analysis of Hub Genes

The correlation between hub gene expression and overall survival was assessed using the Kaplan-Meier estimation method, based on the “survival” package in R. A significant difference in survival curves was assessed using a log-rank test. *P* value less than 0.05 was considered statistically significant.

### Immune Infiltrates Analysis

TIMER^3^ is a database that can comprehensively study the molecular characterization of tumor-immunity interactions. Not only can the association between immune infiltrates and a variety of factors be explored interactively, but also the dynamic analysis and visualization of these associations can be performed using a TIMER. In this study, we evaluated the hub gene expression in GC and its correlation with the abundance of tumor-infiltrating immune cells, via gene modules (Li et al., 2017).

### Molecular Docking

Molecular docking can reflect the binding energetics of drug molecules to protein receptors by calculating the binding affinity between ligands and receptors and the corresponding intermolecular interactions (Huang and Zou, 2010; Ferreira et al., 2015). The crystal structure of the key gene was downloaded from Protein Data Bank (PDB)^4^ database. The 3D protein crystal structure had to be determined by X-ray crystallography and the crystal resolution was less than 3 Å. The protein receptor and ligand files were pre-processed by AutoDock Tools and then Autodock vina was used for molecular docking (Trott and Olson, 2010; Forli et al., 2016). In addition, Pymol and Ligplot were used to visualize the results to show the intermolecular interaction and docking more clearly (Laskowski and Swindells, 2011; Mooers, 2016).

### Cell Lines and Compound Kushen Injection Administration

Human GC cell lines HGC-27 and BGC-823 were purchased from Procell Life Science&Technology Co., Ltd. (Wuhan, China), and cultured in RPMI-1640 medium (Corning, United States) containing 10% fetal bovine serum (Corning, United States) and 1% penicillin/streptomycin (Gibco, United States) in a saturated humidity environment at 37°C and 5% CO2. For CKI incubation (Yang et al., 2020), CKI (total alkaloid concentration of 25 mg/mL) was diluted with culture medium (CKI concentrations: 0.125, 0.25, 0.5, 1.0, 2.0, 4.0, 8.0, 16.0 mg/mL, using the doubling dilution method, based on the total alkaloid concentration in CKI).

### Assessment of the Anticancer Effect of Compound Kushen Injection *in vivo*

The Institute of Hydrobiology, Chinese Academy of Sciences, provided normal AB wild-type zebrafish. The zebrafish were raised and bred under the conditions recommended in *The Zebrafish Book: A guide for the laboratory use of zebrafish (Danio rerio)*. The experiment was in accordance with the Animal Management Rules of the Ministry of Science and Technology of the People’s Republic of China for experimental care and use of animals and approved by the Animal Ethics Committee of Beijing University of Traditional Chinese Medicine.

According to the 1:1 ratio of males to females, healthy adult zebrafish were arranged for natural spawning. Then, zebrafish embryos were collected and cultured in a constant temperature incubator at 28.5°C for the construction of the zebrafish xenograft GC model. Before tumor cell injection, the GC cells HGC-27 were labeled using Cell Plasma Membrane Staining Kit with DiO (Green Fluorescence) (Beyotime, China), and incubated at 37°C in the dark for 20 min. Then the labeled cells were washed with PBS, and digested with trypsin. Subsequently, the cells were resuspended in fresh RPMI-1640 medium (10% FBS, 1% PS) to prepare a cell suspension of 3 × 10^6^ cells/mL liquid. Larva zebrafish at 2 days post fertilization (dpf) were randomly selected for microinjection. The labeled HGC-27 cells were injected into the yolk sac of the zebrafish. After 24 h, the larva zebrafish were placed under a stereofluorescence microscope (Axio Zoom.V16, Carl Zeiss) to observe and image their tumors (63×) at 1 day post-injection (dpi).

The selected tumor-bearing zebrafish were randomly grouped and placed in a 12-well plate with 10 embryos per well. CKI 150, 50, 25, 0 μg/mL (model group) and cisplatin 100 ng/mL (positive control group) were given, respectively. Zebrafish embryo culture water was used for drug preparation and dilution. The treated zebrafish were incubated in a 31°C incubator, and the drug was changed every 24 h. With 4 dpi as the end point of the experiment, the tumor-bearing zebrafish were photographed again with a stereoscopic fluorescence microscope. The tumor fluorescence area and fluorescence intensity in the zebrafish yolk sac area were quantified by Image pro-Plus 6.0 software. Using the tumor fluorescence area and integral optical density (IOD) at 1 dpi as a starting point, the tumor growth rate was calculated.


$$tumourgrowthrate=\frac{IOD\left(4dpi\right)}{IOD\left(1dpi\right)}-1$$

$$tumourgrowthrate=\frac{Area\left(4dpi\right)}{Area\left(1dpi\right)}-1$$

### Cell Viability Assay

The viability of HGC-27 and BGC-823 cells was detected by Cell Counting Kit-8 (CCK-8, Dojindo, Japan) assay. Cells were blown into single cell suspension (0.6 × 10^4^ cells/mL) after routine digestion and seeded in 96-wells plates (100 μL/well), and cultured for 24 h, routinely. Then the cells were cultured with a drug-containing medium for 24, 48, and 72 h, respectively. After the drug treatment, the CCK-8 solution was added into 96-well plates (10 μL/well) and incubated at 37°C for 4 h. The optical density (OD) was detected at 450 nm by using a microplate reader (Molecular Devices, United States).

### Proteomics Analysis

The BGC-823 cells after CKI intervention were used for Tandem Mass Tag (TMT) labeling proteomics and Proteome Discoverer 1.4 software was performed for identification and quantitation analysis. A detailed description of the method was shown in the Supplementary Method 1.

### Real-Time Quantitative PCR Analysis

RNA Easy Fast Cell Kit (Tiangen, China) was applied for total RNA isolation according to the manufacturer’s instruction. The quality of total RNA was accredited by SpectraMax Quick Drop readers (Molecular Devices, United States). Of the total RNA, 1 μg was used for cDNA synthesis following the ReverTra Ace qPCR RT Kit (Toyobo, Japan) instruction. RT-qPCR was performed to measure the relative expression of mRNA, using SYBR Green Realtime PCR Master Mix (Toyobo, Japan). GAPDH was used as a control and the 2^–ΔΔ*Ct*^ method was conducted for the data analysis. The primer sequence of target genes was synthesized as follows: human AKR1B1 (fwd, 5′-CATGCAGAGGAACTTGGTGGTGAT-3′; rev, 5′-TGTTGTAGCTGAGTAAGGTGGTCATATC-3′), human PTGER3 (fwd, 5′-GGTAAACCCAAGGATCCAAGA-3′; rev, 5′-CATCAGTTGAGCACTGCAAGA-3′), human MMP2 (fwd, 5′-TACAGGATCATTGGCTACACACC-3′; rev, 5′-GGTCACAT CGCTCCAGACT-3′), and human GAPDH (fwd, 5′-TGGAGTC CACTGGCGTCTTCAC-3′; rev, 5′-TTGCTGATGATCTTGAG GCTGTTGTC-3′).

### Western Blot Assay

Cells were collected in RIPA lysis buffer and centrifuged at 12000 rpm and 4°C for 20 min. The supernatants were preserved and used for western blot assay. Total protein concentration was gauged by BCA Protein Assay Kit (Solarbio, China). 10 μg of total protein was mixed with 5× sample buffer, boiled at 99°C for 5 min and loaded onto 10% SDS-PAGE gels. Then the protein bands were transferred onto NC membranes and blocked with 5% non-fat milk for 2 h at room temperature. The NC membranes with proteins were incubated with diluted primary antibodies (Proteintech, China) at 4°C overnight, including anti-AKR1B1 (1:1000), anti-PTGER3 (1:1500), anti-MMP2 (1:1000) and anti-GAPDH (1:2000) antibodies. Then, membranes were incubated with relative sources of secondary antibodies (1:5000) at room temperature for 1.5 h. At last, the specific protein bands were recognized with immobilon western chemiluminescent HRP substrate (MilliporeSigma, United States). Image J software was used for image analysis and the signals of specific proteins were normalized to GAPDH or β-Tubulin.

### Statistical Analysis

Data were presented as mean ± SD and statistical analysis was performed with the two-tailed unpaired Student’s *t*-test using GraphPad Prism 8.0 software. In all statistical analyses, statistical significance was pinpointed by a single asterisk (^∗^: *P* < 0.05), two asterisks (^∗∗^: *P* < 0.01).

## Results

### Identification of Major Compounds in Compound Kushen Injection by UPLC-MS

**FIGURE 2:**
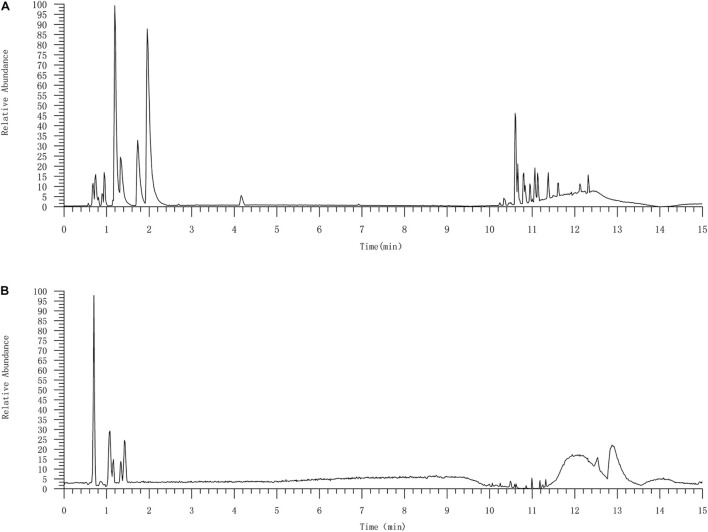
The UPLC-QE-Orbitrap-MS chromatography of CKI **(A)** and standard substances **(B)**. 10 compounds were identified by UPLC-QE-Orbitrap-MS chromatography, including oxysophocarpine, matrine, sophocarpine, sophoridine, oxynamatrine, N-methylcytisine, sophoranol, liriodendrin, trifolirhizin and macrozamin.

In this study, 10 marker ingredients of CKI were identified by UPLC-MS (Figure 2 and Table 1). Simultaneously, we also supplemented the chemical ingredients by a literature research (Ma et al., 2013; Wang et al., 2015). Finally, a total of 16 active ingredients in CKI were selected for the next in-depth study, which included 9α-hydroxymatrine, adenine, baptifoline, isomatrine, lamprolobine, piscidic acid and 10 compounds detected by UPLC-QE-Orbitrap-MS chromatography, and the three-dimensional structures of these active ingredients were derived from PubChem database (Kim et al., 2016).

**TABLE 1:** Identified major compounds in CKI by UPLC-MS.

No.	Rt (min)	Theoretical value	PPM	Molecular Formula	Structure	Rresumption
1	0.729	204.12645	7.03	C15H22N2O2	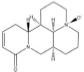	Oxysophocarpine
2	1.075	248.18910	6.88	C15H24N2O	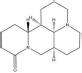	Matrine
3	1.157	246.17360	7.16	C15H22N2O	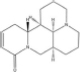	Sophocarpine
4	1.197	248.18896	7.29	C15H24N2O	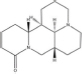	Sophoridine
5	1.970	264.18394	6.66	C15H24N2O2	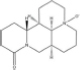	Oxymatrine
6	5.609	218.10577	6.92	C12H16N2O	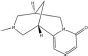	N-methylcytisine
7	7.860	264.18395	6.77	C15H24N2O2	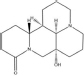	Sophoranol
8	9.920	742.25163	6.31	C34H46O18	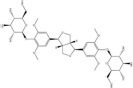	Liriodendrin
9	10.746	446.12215	6.45	C22H22O10	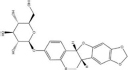	Trifolirhizin
10	11.243	384.22630	2.96	C13H24N2O11	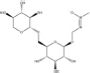	Macrozamin

### Compound Kushen Injection-Predicted Target Network

**FIGURE 3:**
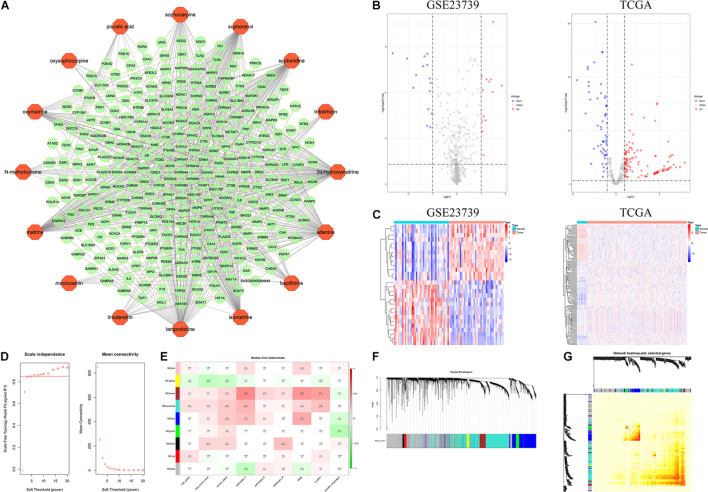
Network pharmacology analysis related to CKI and WGCNA analysis of GC. **(A)** Prediction target network of CKI component. Orange dots indicate the components in CKI, and green dots indicate their predicted targets. **(B)** Volcano map of GSE23739 and TCGA. **(C)** Heat map of GSE23739 and TCGA. **(D)** Soft-thresholding power analysis. *R*^2^ = 0.85. **(E)** Module-trait relationship. Each row corresponds to a ME, and each column corresponds to a clinical trait. Each cell contains a corresponding correlation and *p* value of modules with various clinical traits. **(F)** Clustering dendrogram. corFnc = “pearson”; *power* = 6; minModulesize = 30; mergeCutHeight = 0.2. **(G)** Network TOM heatmap plot.

The active compound-predicted target network (Figure 3A) consists of 301 nodes (16 compound points and 285 gene points) that constitute 636 active compound-predicted target linkages. Details of active compound-predicted targets can be viewed in Supplementary Table 6.

### Screening of Differential MicroRNA in Gastric Carcinoma

In this study, GSE23739 and TCGA were adopted to analyze the DEMs in GC. | log_2_FC| ≥ 1, *P* value < 0.05 and adjust *P* value < 0.05 were considered statistically significant for the DEMs. For GSE23739, a total of 13 up-regulated gene and 15 down-regulated genes were found. Furthermore, there are 107 up-regulated genes and 56 down-regulated genes in the TCGA analysis (Figures 3B,C). Overlapping DEMs (hsa-miR-20a, hsa-miR-30a, hsa-miR-21, hsa-miR-145) between the GSE23739 and TCGA analysis were retained for further study. From miRWALK2.0, 9431 mRNA and 31879 ncRNA targeted by DEMs (hsa-miR-20a-3p, hsa-miR-20a-5p, hsa-miR-30a-3p, hsa-miR-30a-5p, hsa-miR-21-3p, hsa-miR-21-5p, hsa-miR-145-3p, hsa-miR-145-5p) in GC were predicted by more than half of total algorithms (Supplementary Tables 1, 2).

### Construction and Screening of Weighted Gene Co-expression Network Analysis Key Modules

After normalization, no outlier samples were eliminated in the present study. To build a scale-free network, the power of β = 6 (scale free *R*^2^ = 0.85) was chosen as the soft-thresholding parameter (Figures 3D,F). A total of 9 modules were identified via average linkage hierarchical clustering. Clinical traits including vital status, new tumor events, cancer status, pathologic T, pathologic N, pathologic M, stage, *H pylori*, barretts esophagus were selected to calculate the correlation between the module and Pearson test. Evaluated by Pearson test, if *P* < 0.05, the module and clinical characteristics were considered statistically significant. As shown in Figure 3E, the blue, turquoise and brown modules were highly correlated with clinical traits and were identified as key modules. Figure 3G indicated the topological overlap measurement (TOM) heat map of adjacency or topological overlap. TOM plot was made up of randomly selected 400 genes. Each row and column represented a module and the genes of the module. The TOM of co-expressed RNA in the key modules was high, and the internal RNA correlation was also stronger. The network building the key modules was filtered with a weight Cutoff = 0.1 between genes. The blue module consists of 632 genes and 74485 gene linkages. The turquoise module consists of 1239 genes and 126102 gene linkages and the brown module consists of 232 genes and 11316 gene linkages (Supplementary Tables 3–5).

### Construction of Competing Endogenous RNA Network of Compound Kushen Injection Intervention in Gastric Carcinoma

**FIGURE 4:**
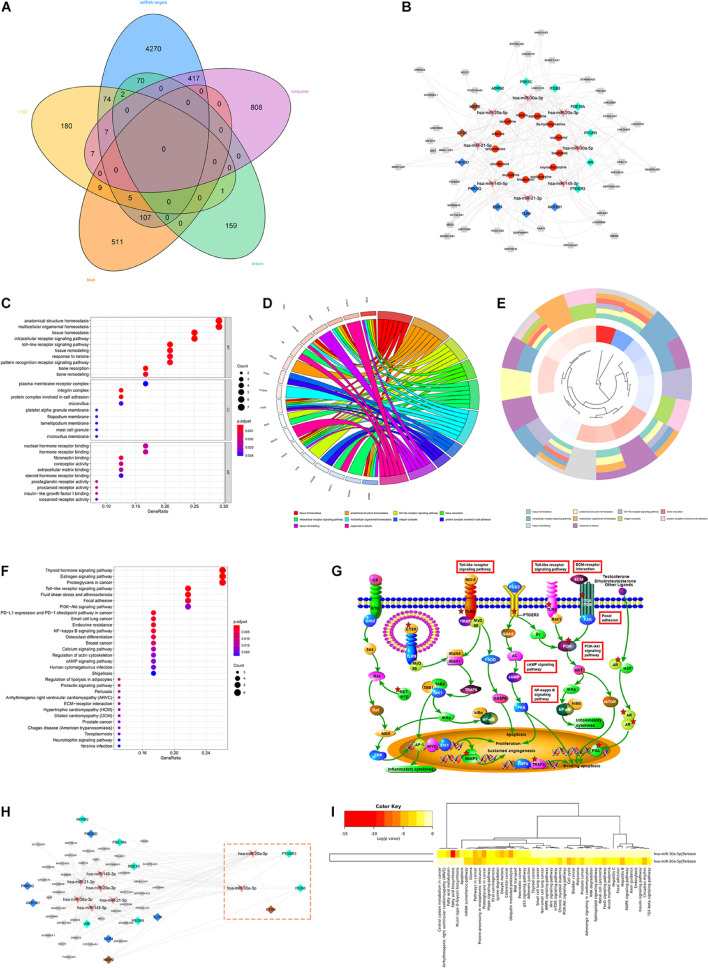
ceRNA network analysis of CKI intervention in GC. **(A)** The Venn diagram of the prediction targets of DEMs and the key modules of WGCNA and the prediction targets of CKI. **(B)** The prediction of ceRNA network of CKI intervention in GC. Orange dots are the components of CKI, and pink arrows indicate miRNAs that may be involved in regulation. Blue, turquoise and brown nodes indicate the key genes of CKI intervention in GC, and the different colors correspond to the module colors in WGCNA. The gray nodes represent the intersection lncRNA in DEMs prediction and WGCNA module. **(C)** The clustering map of the first ten GO pathways. **(D)** GO bubble chart of function enrichment for key genes. **(E)** The circular map of the key genes distribution of the top ten GO pathway. **(F)** KEGG bubble chart of function enrichment for key genes. **(G)** Regulatory pathways mainly involved in the CKI treatment of GC. **(H)** Module analysis of CKI intervention ceRNA. The genes in the pink box constitute the key module. **(I)** Enrichment analysis of key miRNAs pathways.

The intersection of the WGCNA key module network and the hub DEMs prediction target constitutes the ceRNA Network of the lncRNA-miRNA-mRNA Axis in GC. Moreover, the ceRNA network and CKI-predicted target network were merged by Cytoscape to obtain the prediction of the ceRNA network of CKI intervention in GC (Figure 4A). As shown in Figure 4B, the prediction of the ceRNA network of CKI intervention in GC involved 73 nodes and 203 linkages between genes. The overlapping targets (AKR1B1, TLR4, ESR1, PRKCQ, PIK3CD, CTSK, MMP2, ADRB2, PDE1C, ITGB3, PDE10A, PTGFR, AR and PTGER3) were considered as the key genes for the CKI treatment of GC.

### Gene Ontology and Kyoto Encyclopedia of Genes and Genomes Pathway Enrichment Analysis

A total of 14 putative targets were uploaded to the STRING database to identify the functional partnerships and interactions between them. The key genes and their interacting proteins from the PPI network were showed in Supplementary Figure 1. To further interpret the function of the key gene, KEGG and GO annotations were performed in the R software. A total of 127 GO entries were identified, including 93 biological process (BP), 25 molecular function (MF), and 9 cellular component (CC) (*FDR* < 0.01 and *P* < 0.01). The top ten GO terms were tissue homeostasis, anatomical structure homeostasis, toll-like receptor signaling pathway, bone resorption, intracellular receptor signaling pathway, multicellular organismal homeostasis, integrin complex, a protein complex involved in cell adhesion, tissue remodeling, response to ketone (Figures 4C–E). The KEGG results demonstrated that 37 entries satisfy *FDR* < 0.05 and *P* < 0.05 (Figure 4F). These targets were significantly enriched in many pathways related to cancer and signaling pathways, such as the PI3K/AKT signaling pathway. In addition, the Toll-like receptor signaling pathway related to immunity was also significantly enriched (Figure 4G).

We also conducted a modular analysis of the lncRNA-miRNA-mRNA Axis intervened by CKI in Cytoscape by Mcode. A total of key modules were analyzed, including CTSK, ITGB3, PTGER3, hsa-miR-20a-5p and hsa-miR-30a-5p five targets (Figure 4H). To gain insights into the pharmacological mechanisms of CKI on GC, we performed KEGG analysis for two key miRNAs. The results illustrated that the validated targets of hsa-miR-20a-5p and hsa-miR-30a-5p were associated with signaling pathways closely related to cancer and development, such as Pathways in cancer, Hippo signaling pathway and p53 signaling pathway (Figure 4I).

### The Mechanism of Compound Kushen Injection Anti-Gastric Carcinoma in Cellular Proteome

**FIGURE 5:**
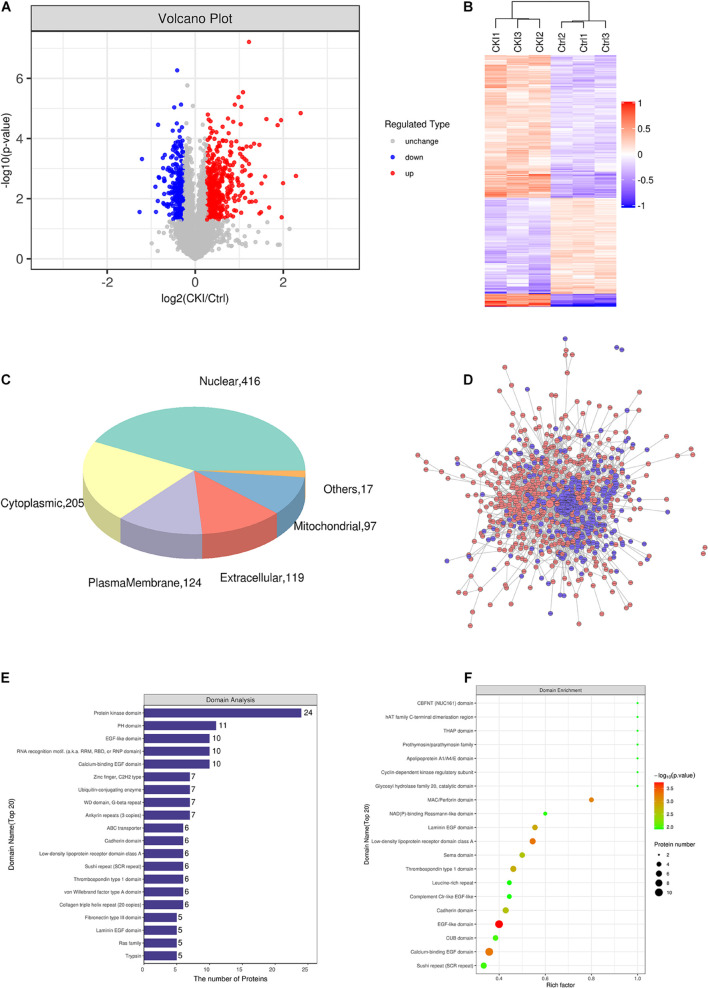
The effects of CKI on proteomics in BGC-823 cells. **(A)** Variance analysis. **(B)** Cluster analysis of DEPs. Total 3 biological replicates for each group were performed. **(C)** Subcellular localization map of DEPs. **(D)** PPI network of DEPs. Red represents up-regulated proteins, and blue means down-regulated proteins. **(E)** Domain analysis of DEPs. **(F)** Domain enrichment analysis.

To further explore the potential mechanism underlying CKI in GC treatment, TMT labeling proteomics was utilized to detect proteome differences between CKI-treated and CKI-untreated GC cells. Quality control, identification and quantification data of proteomics were shown in Supplementary Figure 2. The number of up-regulated DEPs was 490, and down-regulated DEPs was 304 (Figures 5A,B). 210 targets were shared by the proteomic results with those predicted by main active compounds and 561 linkages were attributed to main active compounds and common targets (Supplementary Table 7), which supported the credibility of our network pharmacology results.

Subcellular organelles are micro-organs with specific morphology and function, such as mitochondria, endoplasmic reticulum, etc. They are important sites for proteins to realize various functions. Different subcellular organelles often perform different cellular functions, the analysis of subcellular localization of proteins is helpful to explore the functions of proteins in cells. We found that 416 DEPs were located in the nucleus, 205 DEPs were located in the cytoplasm, 124 DEPs are located in plasma membrane and 119 DEPs were located outside the cells (Figure 5C).

Interactions between proteins and proteins or other ligands with low molecular weight are often based on domains, so domain prediction is of great significance for studying the key functional regions of proteins and their potential bioactivity. Interproscan, a domain prediction software, was used to predict the domain of differentially expressed proteins. Figure 5E shows the first 20 protein domains. And Figure 5F exhibited the domain enrichment characteristics of DEPs, which determine the significance level of protein enrichment.

Proteins, that play a biological regulatory role can interact with other proteins to achieve their function. Systematic analysis of the interaction relationships of a large number of proteins in biological systems is important to understand how proteins work in biological systems, and the response mechanisms of biological signals and metabolism of energy substances in special physiological states, for example, the diseases, and the functional links between proteins. DEPs were brought into the STRING database for PPI annotation, and the PPI network was constructed by Cytoscape software. This network consists of 709 protein nodes and 3125 connections (Figure 5D).

**FIGURE 6:**
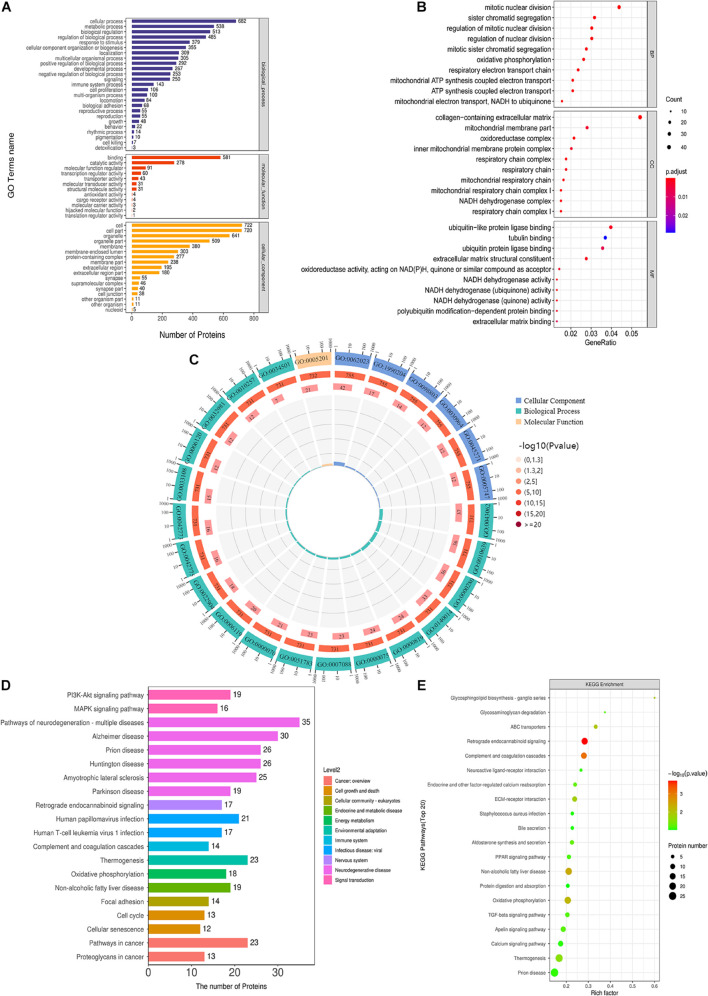
GO and KEGG analysis of DEPs. **(A)** GO annotation of DEPs. **(B)** GO function enrichment bubble diagram of DEPs. **(C)** Top 25 GO entries. **(D)** KEGG pathway annotation statistics of DEPs. **(E)** KEGG pathway enrichment bubble diagram of DEPs.

To comprehensively understand the functions, localization and biological pathways involved in DEPs, GO was used for the biological annotation of proteins. Blast2GO is a high-throughput functional annotation and data mining software. And its features are the combination of various annotation strategies and tools controlling type and intensity of annotation, and the numerous graphical features such as the interactive GO-graph visualization for gene-set function profiling or descriptive charts (Götz et al., 2008). Blast2Go software was utilized to annotate the GO function of DEPs, and counted the number of DEPs at the GO secondary function annotation level (Figure 6A). Fisher’s exact test was applied to analyze the GO functional enrichment of DEPs. The overall functional enrichment characteristics of DEPs were revealed by evaluating the significant level of protein enrichment of a GO functional item. As flashed in Figure 6B, the enrichment analysis revealed that the top five enrichment entries of DEPs were associated with multiple biological processes (BP, including mitotic karyokinesis, regulation of mitotic karyokinesis, sister chromatid separation, mitotic sister chromatid segregation, respiratory electron transport chain), cell compositions (CC, including extracellular matrix containing collagen, mitochondrial respiratory chain complex I, NADH dehydrogenase complex, respiratory chain complex I, oxidoreductase complex) and molecular functions [MF, including structural components of the extracellular matrix, ubiquitin-like protein ligase binding, NADH dehydrogenase activity, NADH dehydrogenase (panquinone) activity, NADH dehydrogenase (quinone) activity]. Figure 6C exhibited the top 25 GO entries (*P* value), including 18 BP entries, 6 CC entries and 1 MF entry.

Kyoto Encyclopedia of Genes and Genomes pathway annotation and enrichment analysis were utilized to clarify the protein-related pathways of DEPs. KEGG Automatic Annotation Server (KAAS) is an online annotation tool provided by KEGG database itself, which is convenient and precise (Moriya et al., 2007). KEGG pathway annotation revealed that DEPs were enriched in cancer-related pathways, cell growth and death-related pathways, such as PI3K-AKT signaling pathway, MAPK signaling pathway, etc. (Figure 6D). KEGG pathway enrichment analysis exhibited that the top 10 pathways (*P* < 0.05) were the retrograde endogenous cannabinin signaling pathway, complement and coagulation cascade pathway, non-alcoholic fatty liver disease pathway, oxidative phosphorylation pathway, ABC transporter pathway, glycosphingolipid biosynthesis-ganglion series pathway, ECM-receptor interaction pathway, fever pathway, aldosterone synthesis and secretion pathway, and glycosaminoglycan degradation pathway (Figure 6E).

### Meta-Analysis of Key Genes

**FIGURE 7:**
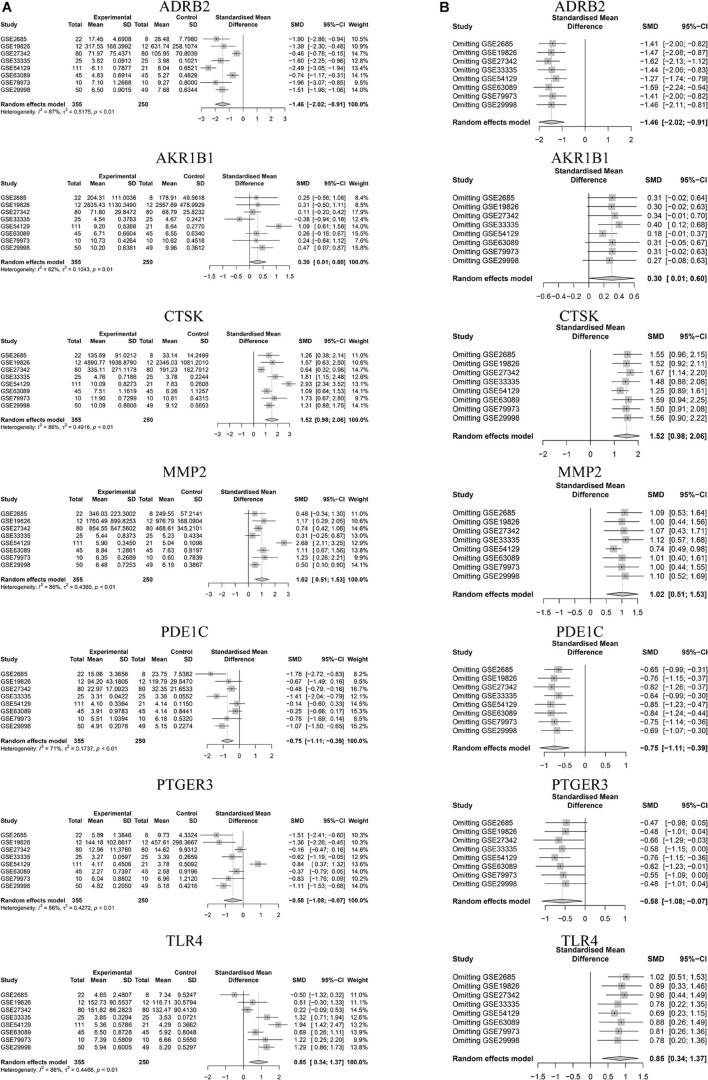
Meta analysis for key genes. **(A)** Forest plot of meta-analysis of key genes. **(B)** Sensitivity analysis of GEO chips of meta-analysis of key genes.

**FIGURE 8:**
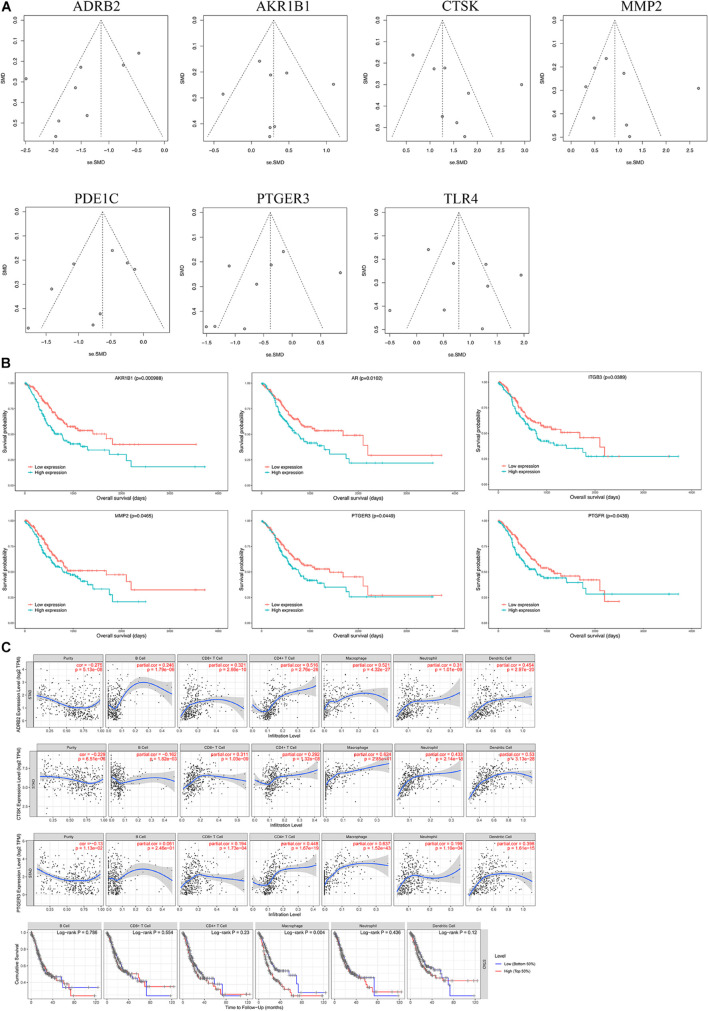
Bioinformatics analysis of key genes. **(A)** A funnel plot was applied to evaluate the publication bias of GEO datasets. **(B)** Survival analysis of key genes. **(C)** Immunoinfiltration analysis.

We selected microarray datasets of gastric cancer tissues from the GEO database for meta-analysis to demonstrate the differential expression of key genes in gastric cancer tissues. A total of 8 microarrays from the GEO database met the entry criteria. The features of the included GEO datasets are depicted in Table 2. The expression data from the tumor and control groups were collected based on the GEO database. Meta-analysis was conducted based on the expression data of the 8 included microarrays. The results (Figure 7A) demonstrated that 7 of the key genes exhibited remarkable abnormal regulation in the gastric cancer groups. Given the apparent heterogeneity, a random effects model was applied. On the one hand, ADRB2 (SMD = −1.46; 95% CI −2.02, − 0.91; *P* < 0.01), PDE1C (SMD = −0.75; 95% CI – 1.11, −0.39; *P* < 0.01) and PTGER3 (SMD = −0.58; 95% CI −1.08; −0.07; *P* < 0.01) were down-regulate in the cancer groups, which might be a tumor suppressor gene. On the other hand, AKR1B1 (SMD = 0.3; 95% CI 0.01; 0.60; *P* < 0.01), CTSK (SMD = 1.52; 95% CI 0.98; 2.06; *P* < 0.01), MMP2 (SMD = 1.02; 95% CI 0.51; 1.53; *P* < 0.01), TLR4 (SMD = 0.85; 95% CI 0.34; 1.37; *P* < 0.01) were up-regulate in cancer groups, which might be the tumor proto-oncogene. Later, a sensitivity analysis was performed to explore whether a particular microarray played a vital role in the significant heterogeneity. It was found that none of the included studies had a decisive role. A funnel plot was generated to estimate publication bias (Figure 7B). The points in the funnel were asymmetrically distributed on both sides of the midline, indicating that the bias was mainly related to publication bias, but there could be other reasons such as the lack of included literature (Figure 8A).

**TABLE 2:** Features of the enrolled Gene Expression Omnibus datasets.

Accession	GPL	Year	GC-count	Control-count	Source
GSE2685	GPL80	2005	22	8	tissue
GSE19826	GPL570	2010	12	12	tissue
GSE27342	GPL5175	2011	80	80	tissue
GSE33335	GPL5175	2012	25	25	tissue
GSE54129	GPL570	2017	111	21	tissue
GSE63089	GPL5175	2014	45	45	tissue
GSE79973	GPL5175	2016	10	10	tissue
GSE29998	GPL6947	2012	50	49	tissue

### Survival Analysis of Key Genes

A Kaplan–Meier curve was later used to identify the effects of hub genes expression on survival time. As shown in Figure 8B, AKR1B1 (*p* = 0.000988), AR (*p* = 0.0102), ITGB3 (*p* = 0.0389), MMP2 (*p* = 0.0465), PTGER3 (*p* = 0.0449), and PTGFR (*p* = 0.0439) with the *p* values were all less than 0.05, suggesting that these genes may be the key targets affecting the survival of GC patients.

### Immunoinfiltration Analysis

Analysis using TIMER showed that hub genes were negatively associated with purity, and ADRB2 (cor = −0.275) was most negatively correlated with tumor purity. In addition, key genes were strongly correlated with macrophages and dendritic cells. Where PTGER3 correlated most strongly with macrophages (cor = 0.637) and CTSK (cor = 0.624) for dendritic cell (Table 3 and Figure 8C). The Univariate Cox survival analysis showed that among the six types of immune cells, only macrophages were associated with survival of GC patients, which was an indicator of survival of GC patients.

**TABLE 3:** Immunoinfiltration analysis of key targets in TIMER.

Gene	Purity	B Cell	CD8+ T Cell	CD4+ T Cell	Macrophage	Neutrophil	Dendritic Cell
ADRB2	−0.275	0.246	0.321	0.516	0.521	0.310	0.454
AKR1B1	−0.100	–0.099	0.337	0.245	0.419	0.359	0.495
AR	−0.143	0.129	0.129	0.484	0.618	0.169	0.352
CTSK	−0.229	–0.162	0.311	0.292	0.624	0.433	0.530
ESR1	−0.216	0.139	0.461	0.608	0.615	0.489	0.621
ITGB3	−0.149	0.134	0.159	0.500	0.471	0.252	0.349
MMP2	−0.182	–0.123	0.183	0.285	0.513	0.273	0.361
PDE10A	−0.115	0.072	0.176	0.306	0.442	0.233	0.340
PDE1C	−0.174	0.303	0.122	0.527	0.432	0.116	0.229
PIK3CD	−0.230	0.065	0.504	0.566	0.409	0.518	0.665
PRKCQ	−0.122	0.031	0.443	0.317	0.239	0.356	0.459
PTGER3	−0.130	0.061	0.194	0.448	0.637	0.199	0.398
PTGFR	−0.192	0.119	0.280	0.486	0.573	0.352	0.445
TLR4	−0.146	–0.121	0.389	0.340	0.548	0.537	0.641

### Molecular Docking

Docking studies were carried out between CKI and hub genes. The 3D protein structures of PTGFR and PDE1C were not found in the PDB database. The molecular docking results are shown in Table 4. AR, ITGB3, AKR1B1, ADRB2, and PTGER3 were five genes with the highest predicted for interaction between each of the five protein targets and corresponding CKI components.

**TABLE 4:** Molecular docking information.

Protein Name	PDB ID	Test Compounds	Affinity (kcal/mol)	Protein Name	PDB ID	Test Compounds	Affinity (kcal/mol)
AKR1B1	4JRI	liriodendrin	−7.8	ITGB3	6BXJ	isomatrine	−6.9
TLR4	5IJD	oxymatrine	−7	ITGB3	6BXJ	lamprolobine	−6.4
ESR1	4XI3	piscidic acid	−6.7	ITGB3	6BXJ	matrine	−7.1
PRKCQ	1XJD	lamprolobine	−7	ITGB3	6BXJ	oxymatrine	−8.2
PIK3CD	5IS5	adenine	−5.2	ITGB3	6BXJ	sophoranol	−7.1
CTSK	2FTD	isomatrine	−6.2	ITGB3	6BXJ	sophoridine	−6.8
CTSK	2FTD	lamprolobine	−6.2	PDE10A	4MVH	9α-hydroxymatrine	−6.2
CTSK	2FTD	matrine	−6.6	PDE10A	4MVH	lamprolobine	−6.4
CTSK	2FTD	oxymatrine	−6.5	PDE10A	4MVH	sophoranol	−6.5
CTSK	2FTD	sophoridine	−6.4	AR	4OEA	9α-hydroxymatrine	−7.5
MMP2	1QIB	adenine	−6.3	AR	4OEA	baptifoline	−8.2
MMP2	1QIB	matrine	−7.3	AR	4OEA	oxysophocarpine	−9.8
ADRB2	3NY9	9α-hydroxymatrine	−7.8	AR	4OEA	sophocarpine	−9.4
ADRB2	3NY9	sophoranol	−6.6	AR	4OEA	sophoranol	−9
PDE1C	1LXS	lamprolobine	−7.8	PTGER3	6AK3	9α-hydroxymatrine	−7.4
ITGB3	6BXJ	9α-hydroxymatrine	−7.3	PTGER3	6AK3	sophoranol	−7.7

**FIGURE 9:**
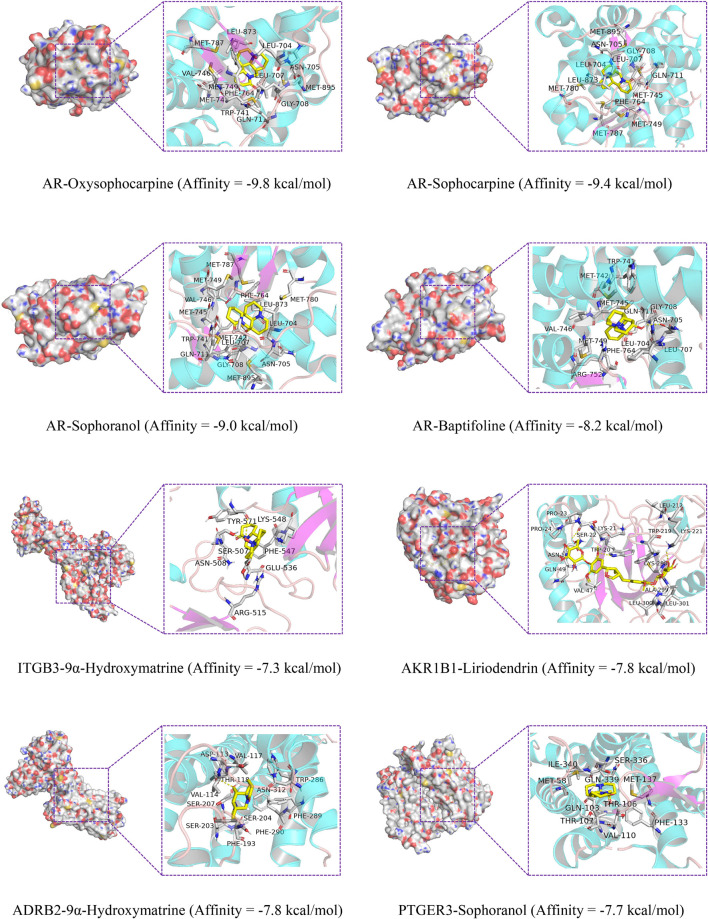
Molecular docking of the key genes with its corresponding component.

As shown in Figure 9, oxysophocarpine binds to a pocket in AR, which consists of Met895, Asn705, Trp741, Leu704, Gly708, Leu707, Gln711, Met745, Met749, Phe764, Val746, Met787, and Leu873. The interaction between AR and sophocarpine is centered on a stable hydrophobic core consisting of several nonpolar residues (Met787, Asn705, Met895, Leu704, Leu707, Gly708, Gln711, Met745, Met749, Phe764, Met780, and Leu873). The fifteen hydrophobic bonds, including Met780, Leu704, Asn705, Met895, Gly708, Leu707, Trp741, Met745, Gln711, Met749, Phe764, Val746, Leu873, Met787, and Met742 are formed in the interaction between sophoranol and AR. Hydrophobic interactions with eleven residues in AR (Leu704, Trp741, Met742, Val746, Gln711, Met749, Phe764, Arg752, Leu707, Met745, and Gly708) and one hydrogen bonds (Asn705). In addition, Oxymatrine can bind to ITGB3 by forming a hydrophobic interaction with the surrounding residues Glu536, Arg515, Asn508, Phe547, Tyr571, and Lys548. Oxymatrine was able to form H-bonds with Tyr571 and Ser507.

The docking results in this study demonstrate that the receptor–ligand interaction between liriodendrin and AKR1B1 involves both hydrophobic and polar interactions. Their interaction is centered on a stable hydrophobic core consisting of several nonpolar residues in AKR1B1 (Pro24, Gln49, Ala212, Lys21, Val47, Trp20, Cys298, Leu301, Trp219, Pro23, and Asn50). Moreover, the hydroxyl groups within the main chains of Leu300, Ala299, Lys211, Trp20, and Ser22 form five hydrogen bonding contacts with the liriodendrin, which further stabilizes the entire interaction region. And 9α-hydroxymatrine was observed to form hydrophobic interactions with eleven residues in ADRB2 (Phe290, Ser203, Ser204, Phe289, Phe193, Asn312, Asp113, Val117, Val114, Trp286, and Ser207) and one hydrogen bonds with Thr118. The fifth genes PTGER3 bind to sophoranol with ten hydrophobic bonds, including Gln339, Ser336, Met58, Ile340, Gln103, Thr107, Val110, Met137, Phe133, and Thr106.

### Anti-Gastric Carcinoma Effect of Compound Kushen Injection

**FIGURE 10:**
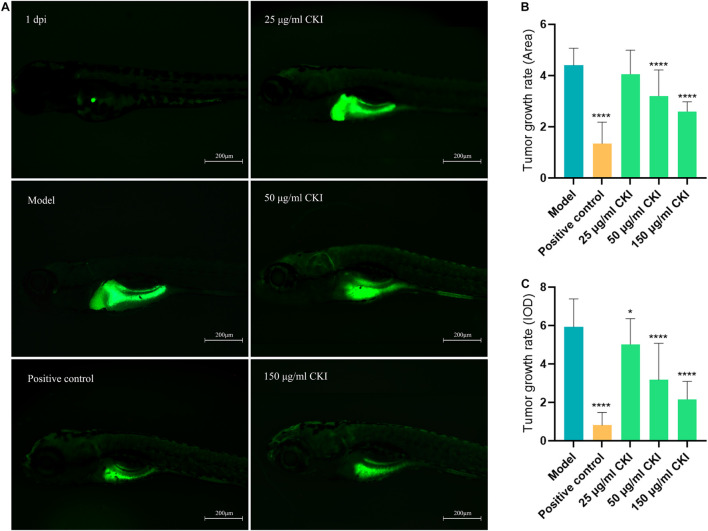
Anti-tumor proliferation effect of CKI in zebrafish. **(A)** Tumor growth exhibited by DiO fluorescence. **(B)** Tumor growth rate calculated by DiO fluorescence Area. **(C)** Tumor growth rate calculated by DiO fluorescence IOD. Data were presented as mean ± SD. *n* = 24. *P < 0.05; ****P < 0.0001.

**FIGURE 11:**
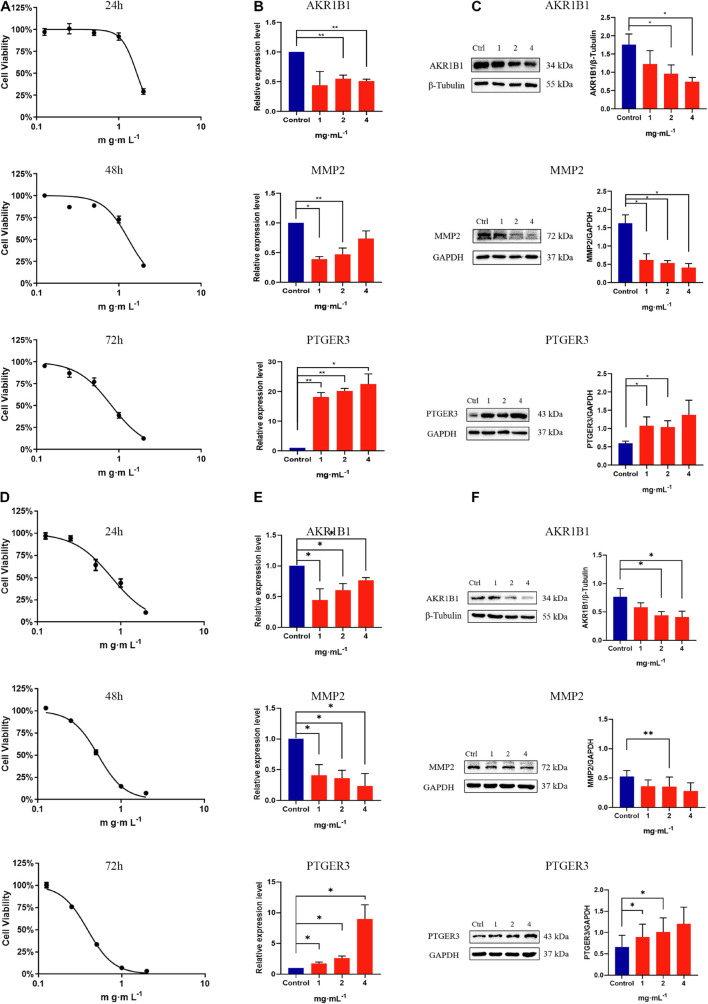
The expressions of AKR1B1, MMP2, and PTGER3 were detected in GC cells. In BGC-823 cells, **(A)** Dose-inhibition curves of CKI. For 24 h, IC_50_ = 1.72 ± 0.22 mg/mL; for 48 h, IC_50_ = 1.20 ± 0.11 mg/mL; for 72 h, IC_50_ = 0.79 ± 0.05 mg/mL. **(B)** mRNA level of key genes was measured by RT-qPCR after CKI intervention. **(C)** Protein level of key genes was determined by western blot after CKI intervention. In HGC-27 cells, **(D)** Dose-inhibition curves of CKI. For 24 h, IC_50_ = 0.98 ± 0.17 mg/mL; for 48 h, IC_50_ = 0.64 ± 0.13 mg/mL; for 72 h, IC_50_ = 0.62 ± 0.22 mg/mL. **(E)** mRNA level of key genes was measured by RT-qPCR after CKI intervention. **(F)** Protein level of key genes was determined by western blot after CKI intervention. Data were presented as mean ± SD. *n* = 3. ^∗^*P* < 0.05; ^∗∗^*P* < 0.01.

Zebrafish has great advantages in real-time monitoring of tumor cell growth *in vivo* and is widely used in human cancer research through the tumor xenograft technique. In this study, different concentrations of CKI and the positive drug cisplatin were used to treat tumor-bearing zebrafish. After 72 h of continuous exposure to the drug, the fluorescence area and intensity of the tumor in the yolk sac area of the low-, medium-, and high-dose groups and the positive control group were decreased, compared with the model group, indicating that 25–150 μg/mL CKI had an inhibitory effect on the growth of the transplanted tumor (Figure 10). And the inhibitory effect was concentration-dependent. Figure 11A, showed that CKI treatment had no significant inhibitory effect for 24 h, except for the 2.0 mg/mL group. At 48 h, 1.0 and 2.0 mg/mL had a significant inhibitory effects on BGC-823 cells. After 72 h, concentrations above 0.5 mg/mL showed significant inhibitory effects. As shown in Figure 11D, the concentration of CKI reached above 0.25 mg/mL, it began to produce apparent inhibitory effect on cell proliferation of HGC-27 cells. The inhibitory effect on cell proliferation gradually increased with the increase of drug concentration.

### Effect of Compound Kushen Injection on the Expression of AKR1B1, MMP2, PTGER3 in Gastric Carcinoma Cells

BGC-823 and HGC-27 cells were used to analyze the regulatory effects of CKI on key genes (AKR1B1, MMP2, and PTGER3) in GC cells to evaluate the mechanism of CKI in GC treatment. After 48 h of CKI treatment, compared with the control group in BGC-823 cells (Figure 11B), mRNA expression levels of AKR1B1 decreased significantly in CKI 2.0 and 4.0 mg/mL groups (*P* < 0.05), MMP2 declined significantly in CKI 1.0 and 2.0 mg/mL groups (*P* < 0.05), and PTGER3 increased significantly in all CKI groups (*P* < 0.05). Similarly, we obtained the same validation in HGC-27 cells (Figure 11E). To further confirm the regulatory effects of CKI on key genes (AKR1B1, MMP2, and PTGER3), a western blot assay was used to detect the extent of protein variation intervened by CKI in BGC-823 and HGC-27 cells. After 48 h of CKI treatment, protein expression levels of AKR1B1 were significantly reduced (*P* < 0.05) in BGC-823 cells compared with the control group (Figure 11C) in the 2.0 and 4.0 mg/mL CKI groups, MMP2 was significantly decreased (*P* < 0.05) in all CKI groups, and PTGER3 was significantly increased (*P* < 0.05) in the 1.0 and 2.0 mg/mL CKI groups. Approximately, we obtained the comparable validation in HGC-27 cells (Figure 11F).

## Discussion

Gastric cancer is one of the most common cancers and its mortality rate remains high (Waldum et al., 2017). Since GC is difficult to detect in the early stages, there is always a delay in diagnosis in patients with gastric cancer (Zhang L. et al., 2019). Therefore, due to the crisis situation of GC with low chance of cure and poor prognosis, there is an urgent need to develop new therapies. CKI is a prescribed drug approved by the Chinese Medicine Administration of China (NMPA). It has also passed the standardized Good Manufacturing Process (GMP) certification and is widely used clinically to treat gastric cancer (Zhao et al., 2014). According to several systematic reviews and meta-analysis studies, it has been found that CKI can not only improve the clinical efficacy in gastric cancer patients but also mitigate the adverse effects of radiotherapy and chemotherapy (Zhang et al., 2017, 2018; Huang and Wei, 2019).

Cancer is a complex disease that results from changes in multiple biological networks (Pache et al., 2012). Therefore, in this study, we established an advanced systems pharmacology strategy that was used to reveal the mechanism underlying the effects of CKI on GC. In detail, this initiative strategy was integrated WGCNA, molecular target prediction technology, microarray analysis, meta-analysis, molecular docking technology, proteomics and experimental verification *in vivo* and *in vitro*. The high-throughput data analysis method was used to find miRNAs closely associated with gastric cancer for target prediction and overlapped with the key modules of WGCNA analysis in TCGA to identify ceRNA networks closely related to GC. Finally, considering network pharmacology as a core concept, the key target of CKI in gastric cancer was identified, and 14 intersection genes were identified as hub genes. To further explore the impact of CKI on gastric cancer, we conducted a meta-analysis of key targets to compare the differential expression of key genes in gastric cancer tissues and normal tissues. Second, we performed functional analysis to understand the biological regulatory pathways involved in key genes. In addition, survival analysis and immune infiltration analysis were used to analyze the relationship between key genes and the prognosis of gastric cancer. Finally, molecular docking simulation was used to verify the binding of CKI components to key targets.

Network pharmacology can explain the effects of drugs on the disruptions of biological networks from the perspective of macro or overall regulation, and explain the treatment of diseases from the perspective of multi-component-multi-target-multi-pathway (Jing et al., 2019). We discovered through network pharmacology that 14 intersection genes could be the key targets for CKI treatment of GC. After meta-analysis of GC gene expression profile chip, it was found that 7 of these genes, including AKR1B1, CTSK, MMP2, TLR4, ADRB2, PDE1C, and PTGER3, had significant differences in gastric cancer tissues. Besides, AKR1B1, MMP2 and PTGER3 were found to be important in the analysis of gastric cancer survival, so the above three genes are considered to be the most significant hub genes for CKI to treat gastric cancer and improve the prognosis of GC. AKR1B1 as a common high-expressed gene in cancer, including gastric cancer, may lead to increased proliferation, metastasis and invasion of tumor cells by driving the epithelial-tomesenchymal transition (EMT) (Wu et al., 2017; Schwab et al., 2018). Studies have shown that ADRB2 can directly interact with and upregulate AKR1B1 in pancreatic cancer cells, promoting cell proliferation and inhibiting apoptosis through the ERK1/2 pathway (Xiao et al., 2018). In addition, the expression of MMP2 in AKR1B1 knockdown cancer cells also decreased significantly compared with the control group (Schwab et al., 2018). MMP2 has been implicated in tumor development and morphogenesis (Zhao et al., 2019). It has been demonstrated that MMP-2 can regulate the extracellular matrix (ECM) degradation, which plays an important role in cancer development (Zhang et al., 2012). Previous studies have confirmed that matrine, an important component of CKI, can downregulate the abnormal expression of MMP2 and thus inhibit tumor cell invasion and metastasis (Qian et al., 2015; Huang et al., 2017; Gao et al., 2018). Whole-genome analysis showed that PTGER3 is abnormally low in gastric cancer. PTGER3 can inhibit the secretion of gastric parietal cells and gastric acid (Kim et al., 2018). The lack of PTGER3 leads to abnormal secretion of gastrin and gastric acid and accelerates the occurrence of gastric cancer (Nishio et al., 2007). In addition, PTGER3 can also up-regulate the expression of related MMP2 and AR to promote the proliferation of cancer cells and the deterioration of gastric cancer (Robertson et al., 2010; Kashiwagi et al., 2013). Indeed, we also found that CKI could induce the changes of intracellular AKR1B1, MMP2 and PTGER3 at mRNA and protein levels in GC cell lines BGC-823 and HGC-27, and the change trends were consistent in these two levels. Therefore, we consider that these may be important approaches of CKI treatment of GC.

In this study, we performed GO enrichment and KEGG pathway analysis to elucidate the multiple mechanisms of CKI against GC from a systematic level. The key genes of CKI on GC were enriched in the PI3K/AKT signaling pathway, Toll-like receptor signaling pathway and other pathways, as indicated by the functional enrichment analysis. With frequent alterations identified in GC, the PI3K/AKT pathway is significantly involved in gastric carcinogenesis and progression (Matsuoka and Yashiro, 2014). The PI3K/AKT pathway can be activated by various factors, including hormone and ECM signaling pathways, thereby regulating several basic cellular activities such as cell proliferation, apoptosis and metastasis (Fresno et al., 2004). As the most common dysregulatory pathway in cancer, the PI3K/AKT pathway has received increasing attention due to its potential for targeted therapy in many malignancies (Hu et al., 2019). We proposed that CKI plays a therapeutic role in the treatment of GC mediated by the PI3K/AKT pathway, and this has been previously confirmed experimentally. Previous studies have confirmed that the active ingredients of matrine, oxymatrine and sophoridine in CKI can treat various tumors by inhibiting the PI3K/AKT signaling pathway (Wang et al., 2017; Dai et al., 2018; Zhang X. et al., 2019). Peng et al. (2016) found that matrine can inhibit the proliferation and metastasis of gastric cancer cell SGC7901 via PI3K/Akt pathway. Accordingly, by comparing the proteomics results between the CKI group and the control group, we found that DEPs were significantly enriched in the PI3K/AKT signaling pathway, MAPK signaling pathway, ABC transporter pathway, ECM receptor interaction pathway and other signaling pathways. Excessive activation of the PI3K/AKT signaling pathway leads to over-expression of P-gp (ABC carrier), which affects the normal function of the ABC transporter signaling pathway (Sui et al., 2014). Abnormal ABC transporter pathway is common in multidrug resistance. MAPKs transduce signals through tertiary kinase cascades, and activated MAPKs maintain normal cellular functioning in the nucleus (Meister et al., 2013). MAPK subgroups, including ERK and JNK, and their signaling pathways are intimately associated with the regulation of ABC transporters, affecting multidrug resistance (Huang et al., 2014; Ji et al., 2015). Accordingly, we speculate that PI3K/AKT, MAPK, ABC transporters, ECM-receptor interaction pathways and the crosstalk between them are the potential mechanisms of CKI that regulate the basic activities (proliferation, migration, invasion) of GC cells and reverse the drug resistance of GC cells.

From a comprehensive analysis of the above results, we also found that immunization may be an important potential influencing factor in CKI treatment of GC, therefore we performed an analysis of the immune invasion of key genes for gastric cancer. The analysis revealed that the degree of macrophage infiltration affects the prognosis of GC patients, and the expression of key genes is positively correlated with the degree of macrophage infiltration. Consistent with this, sophoridine has been shown to polarize tumor-associated macrophages (TAMs) into M1-TAMs and suppress M2-TAMs polarization through the TLR4/IRF3 axis. In addition, it can inhibit the migration ability of macrophages and reshape the immunological microenvironment of gastric cancer (Zhuang et al., 2020). Taken together, we propose that CKI can not only directly inhibit the proliferation and metastasis of gastric cancer tumor cells, but also improve the prognosis of cancer patients through immunotherapy.

In addition to the key mRNAs, we found two significant miRNAs possibly involved in CKI regulation of gastric cancer, namely hsa-miR-20a-5p and hsa-miR-30a-5p, by module analysis. KEGG pathway analysis of miRNA’s demonstrated that most of the target genes were enriched in the Hippo signaling pathway and p53 signaling pathway, which are closely associated with cancer cell proliferation and metastasis. Matrine plays an important role in regulating the p53 signaling pathway to inhibit cell proliferation in liver cancer, lung cancer and esophageal cancer (Wang et al., 2014; Xie et al., 2015; Lu et al., 2017). Besides, matrine can also promote apoptosis of colorectal cancer cells via the Hippo signaling pathway (Zhang Y. et al., 2019). It is worth mentioning that the study found that sophoridine can significantly activate the Hippo and p53 signaling pathways and inhibit the progression of lung cancer and enhance the effect of the anticancer drug cisplatin against lung cancer cells (Zhu et al., 2020). However, there are few studies on the direct effect of CKI and its active ingredients on miRNA in the treatment of gastric cancer, which needs to be confirmed by experiments *in vivo* and *in vitro*.

## Conclusion

This study explored the potential molecular mechanism of CKI in the treatment of GC and established a new advanced system pharmacology strategy. Based on the traditional network pharmacology, the potential molecular mechanism of CKI in the treatment of GC was initially determined by the analysis of high-throughput chip data and WGCNA. Moreover, chip meta-analysis methods and survival analysis were used to verify the expression and prognosis of key genes in GC. Functional enrichment analysis and immune infiltration analysis focus on the functional impact of key genes. Finally, molecular docking was employed to verify the strong binding between the target and the components. By using network pharmacology combined with multiple integrated bioinformatics methods, we systematically revealed that CKI might be involved in regulating the ceRNA network for the treatment of GC. Among them, mRNA (including AKR1B1, MMP2 and PTGER3) and miRNA (including hsa-miR-20a-5p and hsa-miR-30a-5p) may play an essential role in the therapy. Our preliminary conclusion is that CKI could be used for GC therapy by activating signaling pathways such as PI3K/AKT, MAPK, ABC transporter and ECM-receptor interaction pathways to inhibit cancer cell proliferation and regulate immunity. Based on a multidisciplinary approach, this study might provide a new perspective for the profound exploration and provide a reference for multicomponent-multitarget-multipathway clinical research.

## Data Availability Statement

The datasets presented in this study can be found in online repositories. The names of the repository/repositories and accession number(s) can be found in the article/Supplementary Material.

## Ethics Statement

The experiment was in accordance with the Animal Management Rules of the Ministry of Science and Technology of the People’s Republic of China for experimental care and use of animals and approved by the Animal Ethics Committee of Beijing University of Traditional Chinese Medicine.

## Author Contributions

WZ, JW, and RY designed the experiments and wrote the manuscript. WZ and XL collected and analyzed the bioinformatics data. CW, CZ, ZH, and XF performed most of the experiments. SL and ZW interpreted the experimental data and visualized the results. YT, AS, and JZ substantively revised the manuscript. All authors gave the final approval of the version to be published.

## Conflict of Interest

RY was employed by the company Shanxi Zhendong Pharmaceutical Co., Ltd. The authors declare that this study received funding from Shanxi Zhendong Pharmaceutical Co., Ltd. The funder had the following involvement with the study: Study design. The remaining authors declare that the research was conducted in the absence of any commercial or financial relationships that could be construed as a potential conflict of interest.

## Publisher’s Note

All claims expressed in this article are solely those of the authors and do not necessarily represent those of their affiliated organizations, or those of the publisher, the editors and the reviewers. Any product that may be evaluated in this article, or claim that may be made by its manufacturer, is not guaranteed or endorsed by the publisher.

## Footnotes

1
http://www.cytoscape.org/

2
https://string-db.org/

3
https://cistrome.shinyapps.io/timer/

4
https://www.rcsb.org/

## Supplementary Material

Supplementary Figure 1 PPI network of key genes.

Supplementary Figure 2 Quality control **(A–C)**, identification and quantification **(D)** of proteomics.

Supplementary Table 1 mRNA targets predicted by miRNA.

Supplementary Table 2 ncRNA targets predicted by miRNA.

Supplementary Table 3 Blue network nodes in WGCNA.

Supplementary Table 4 Brown network nodes in WGCNA.

Supplementary Table 5 Turquoise network nodes in WGCNA.

Supplementary Table 6 Active compound-predicted targets.

Supplementary Table 7 Common targets.

Supplementary Method 1 Proteomics analysis.

## Abbreviations

ceRNA: competing endogenous RNA
CI: confidential interval
CKI: Compound Kushen injection
DEMs: differentially expressed miRNAs
ECM: extracellular matrix
EMT: epithelial-tomesenchymal transition
GC: gastric carcinoma
GEO: Gene Expression Omnibus
GMP: Good Manufacturing Process
GO: Gene Ontology
GS: Gene Significance
KEGG: Functional and Kyoto Encyclopedia of Genes and Genomes
lncRNA: long noncoding RNA
M: mean
ME: Module eigengene
miRNA: microRNA
MM: Module Membership
ncRNA: noncoding RNA
NMPA: Chinese Medicine Administration of China
PDB: Protein Data Bank
SD: standard deviation
STITCH: Search Tool for Interactions of Chemicals
SMD: standardized meta-difference
STRING: Search Tool for the Retrieval of Interacting Genes/Proteins
PPI: protein-protein interaction
TAMs: tumor-associated macrophages
TCGA: The Cancer Genome Atlas
TCMSP: Traditional Chinese Medicine Systems Pharmacology Database and Analysis Platform
TOM: Topological Overlap Measure
WGCNA: weighted gene co-expression network analysis.

## References

[B1] Ajani J. A. Lee J. Sano T. Janjigian Y. Y. Fan D. Song S. (2017). Gastric adenocarcinoma. *Nat. Rev. Dis. Primes* 3:17036. 10.1038/nrdp.2017.36 28569272

[B2] Bray F. Ferlay J. Soerjomataram I. Siegel R. L. Torre L. A. Jemal A. (2018). Global cancer statistics 2018: globocan estimates of incidence and mortality worldwide for 36 cancers in 185 countries. *CA Cancer J. Clin.* 68 394–424. 10.3322/caac.21492 30207593

[B3] Cancer Genome Atlas Research Network (2014). Comprehensive molecular characterization of gastric adenocarcinoma. *Nature* 513 202–209. 10.1038/nature13480 25079317 PMC4170219

[B4] Coccolini F. (2016). Advanced gastric cancer: what we know and what we still have to learn. *World J. Gastroentero.* 22:1139. 10.3748/wjg.v22.i3.1139 26811653 PMC4716026

[B5] Dai Z. Wang L. Wang X. Zhao B. Zhao W. Bhardwaj S. S. (2018). Oxymatrine induces cell cycle arrest and apoptosis and suppresses the invasion of human glioblastoma cells through the egfr/pi3k/akt/mtor signaling pathway and stat3. *Oncol. Rep.* 40 867–876. 10.3892/or.2018.6512 29989652

[B6] Dweep H. Gretz N. (2015). Mirwalk2.0: a comprehensive atlas of microrna-target interactions. *Nat. Methods.* 12:697. 10.1038/nmeth.3485 26226356

[B7] Ferreira L. G. Dos S. R. Oliva G. Andricopulo A. D. (2015). Molecular docking and structure-based drug design strategies. *Molecules* 20 13384–13421. 10.3390/molecules200713384 26205061 PMC6332083

[B8] Ferro A. Peleteiro B. Malvezzi M. Bosetti C. Bertuccio P. Levi F. (2014). Worldwide trends in gastric cancer mortality (1980–2011), with predictions to 2015, and incidence by subtype. *Eur. J. Cancer.* 50 1330–1344. 10.1016/j.ejca.2014.01.029 24650579

[B9] Forli S. Huey R. Pique M. E. Sanner M. F. Goodsell D. S. Olson A. J. (2016). Computational protein-ligand docking and virtual drug screening with the autodock suite. *Nat. Protoc.* 11 905–919. 10.1038/nprot.2016.051 27077332 PMC4868550

[B10] Franz M. Lopes C. T. Huck G. Dong Y. Sumer O. Bader G. D. (2016). Cytoscape.js: a graph theory library for visualisation and analysis. *Bioinformatics* 32 309–311. 10.1093/bioinformatics/btv557 26415722 PMC4708103

[B11] Fresno V. J. Casado E. de Castro J. Cejas P. Belda-Iniesta C. Gonzalez-Baron M. (2004). Pi3k/akt signalling pathway and cancer. *Cancer Treat. Rev.* 30 193–204. 10.1016/j.ctrv.2003.07.007 15023437

[B12] Gao L. Wang K. X. Zhou Y. Z. Fang J. S. Qin X. M. Du G. H. (2018). Uncovering the anticancer mechanism of compound kushen injection against hcc by integrating quantitative analysis, network analysis and experimental validation. *Sci. Rep.* 8:624. 10.1038/s41598-017-18325-7 29330507 PMC5766629

[B13] Gfeller D. Grosdidier A. Wirth M. Daina A. Michielin O. Zoete V. (2014). Swisstargetprediction: a web server for target prediction of bioactive small molecules. *Nucleic Acids Res.* 42 W32–W38. 10.1093/nar/gku293 24792161 PMC4086140

[B14] Götz S. García-Gómez J. Terol J. Williams T. Nagaraj S. Nueda M. (2008). High-throughput functional annotation and data mining with the Blast2GO suite. *Nucleic Acids Res.* 36 3420–3435. 10.1093/nar/gkn176 18445632 PMC2425479

[B15] Hu M. Zhu S. Xiong S. Xue X. Zhou X. (2019). Micrornas and the pten/pi3k/akt pathway in gastric cancer (review). *Oncol. Rep.* 41 1439–1454. 10.3892/or.2019.6962 30628706

[B16] Huang C. Xu D. Ju J. Xia Q. Wang M. (2014). The regulatory mechanism of JNK signal transduction pathway-mediated multidrug-resistance in human hepatic cancer cell line Bel-7402/FU. *Tumor* 34 19–25.

[B17] Huang H. Du T. Xu G. Lai Y. Fan X. Chen X. (2017). Matrine suppresses invasion of castration-resistant prostate cancer cells by downregulating mmp-2/9 via nf-kappab signaling pathway. *Int. J. Oncol.* 50 640–648. 10.3892/ijo.2016.3805 28000853 PMC6903897

[B18] Huang S. Y. Zou X. (2010). Advances and challenges in protein-ligand docking. *Int. J. Mol. Sci.* 11 3016–3034. 10.3390/ijms11083016 21152288 PMC2996748

[B19] Huang Z. Wei P. (2019). Compound kushen injection for gastric cancer: a protocol of systematic review and meta-analysis. *Medicine (Baltimore)* 98:e17927. 10.1097/MD.0000000000017927 31702676 PMC6855605

[B20] Jeong H. Mason S. P. Barabasi A. L. Oltvai Z. N. (2001). Lethality and centrality in protein networks. *Nature* 411 41–42. 10.1038/35075138 11333967

[B21] Ji R. Zhang B. Zhang X. Xue J. Yuan X. Yan Y. (2015). Exosomes derived from human mesenchymal stem cells confer drug resistance in gastric cancer. *Cell Cycle* 14 2473–2483. 10.1080/15384101.2015.1005530 26091251 PMC4613597

[B22] Jing C. Sun Z. Xie X. Zhang X. Wu S. Guo K. (2019). Network pharmacology-based identification of the key mechanism of qinghuo rougan formula acting on uveitis. *Biomed. Pharmacother.* 120:109381. 10.1016/j.biopha.2019.109381 31542616

[B23] Kashiwagi E. Shiota M. Yokomizo A. Itsumi M. Inokuchi J. Uchiumi T. (2013). Prostaglandin receptor ep3 mediates growth inhibitory effect of aspirin through androgen receptor and contributes to castration resistance in prostate cancer cells. *Endocr. Relat. Cancer* 20 431–441. 10.1530/ERC-12-0344 23493387

[B24] Kim H. J. Kang T. W. Haam K. Kim M. Kim S. K. Kim S. Y. (2018). Whole genome mbd-seq and rrbs analyses reveal that hypermethylation of gastrointestinal hormone receptors is associated with gastric carcinogenesis. *Exp. Mol. Med.* 50 1–14. 10.1038/s12276-018-0179-x 30510283 PMC6277407

[B25] Kim S. Thiessen P. A. Bolton E. E. Chen J. Fu G. Gindulyte A. (2016). Pubchem substance and compound databases. *Nucleic Acids Res.* 44 D1202–D1213. 10.1093/nar/gkv951 26400175 PMC4702940

[B26] Langfelder P. Horvath S. (2008). Wgcna: an r package for weighted correlation network analysis. *BMC Bioinformatics.* 9:559. 10.1186/1471-2105-9-559 19114008 PMC2631488

[B27] Laskowski R. A. Swindells M. B. (2011). Ligplot+: multiple ligand-protein interaction diagrams for drug discovery. *J. Chem. Inf. Model.* 51 2778–2786. 10.1021/ci200227u 21919503

[B28] Li A. Horvath S. (2007). Network neighborhood analysis with the multi-node topological overlap measure. *Bioinformatics* 23 222–231. 10.1093/bioinformatics/btl581 17110366

[B29] Li T. Fan J. Wang B. Traugh N. Chen Q. Liu J. S. (2017). Timer: a web server for comprehensive analysis of tumor-infiltrating immune cells. *Cancer Res.* 77 e108–e110. 10.1158/0008-5472.CAN-17-0307 29092952 PMC6042652

[B30] Liu H. Zhang Q. Lou Q. Zhang X. Cui Y. Wang P. (2020). Differential analysis of lncrna, mirna and mrna expression profiles and the prognostic value of lncrna in esophageal cancer. *Pathol. Oncol. Res.* 26 1029–1039. 10.1007/s12253-019-00655-8 30972633

[B31] Liu Y. Zhu J. Ma X. Han S. Xiao D. Jia Y. (2019). Cerna network construction and comparison of gastric cancer with or without *helicobacter* pylori infection. *J. Cell. Physiol.* 234 7128–7140. 10.1002/jcp.27467 30370523

[B32] Lu Z. Xiao Y. Liu X. Zhang Z. Xiao F. Bi Y. (2017). Matrine reduces the proliferation of a549 cells via the p53/p21/pcna/eif4e signaling pathway. *Mol. Med. Rep.* 15 2415–2422. 10.3892/mmr.2017.6331 28447756 PMC5428535

[B33] Ma Y. Gao H. Liu J. Chen L. Zhang Q. Wang Z. (2013). Identification and determination of the chemical constituents in a herbal preparation, compound kushen injection, by hplc and lc-dad-ms/ms. *J. Liq. Chromatogr. Relat. Technol.* 37 207–220. 10.1080/10826076.2012.738623

[B34] Matsuoka T. Yashiro M. (2014). The role of pi3k/akt/mtor signaling in gastric carcinoma. *Cancers (Basel)* 6 1441–1463. 10.3390/cancers6031441 25003395 PMC4190549

[B35] Meister M. Tomasovic A. Banning A. Tikkanen R. (2013). Mitogen-activated protein (map) kinase scaffolding proteins: a recount. *Int. J. Mol. Sci.* 14 4854–4884. 10.3390/ijms14034854 23455463 PMC3634400

[B36] Mooers B. H. (2016). Simplifying and enhancing the use of pymol with horizontal scripts. *Protein Sci.* 25 1873–1882. 10.1002/pro.2996 27488983 PMC5029532

[B37] Moriya Y. Itoh M. Okuda S. Yoshizawa A. Kanehisa M. (2007). KAAS: an automatic genome annotation and pathway reconstruction server. *Nucleic Acids Res.* 35 182–185. 10.1093/nar/gkm321 17526522 PMC1933193

[B38] Nickel J. Gohlke B. O. Erehman J. Banerjee P. Rong W. W. Goede A. (2014). Superpred: update on drug classification and target prediction. *Nucleic Acids Res.* 42 W26–W31. 10.1093/nar/gku477 24878925 PMC4086135

[B39] Nishio H. Terashima S. Nakashima M. Aihara E. Takeuchi K. (2007). Involvement of prostaglandin e receptor ep3 subtype and prostacyclin ip receptor in decreased acid response in damaged stomach. *J. Physiol. Pharmacol.* 58 407–421. 17928639

[B40] Pache R. A. Ceol A. Aloy P. (2012). Netaligner–a network alignment server to compare complexes, pathways and whole interactomes. *Nucleic Acids Res.* 40 W157–W161. 10.1093/nar/gks446 22618871 PMC3394252

[B41] Paraskevopoulou M. D. Vlachos I. S. Hatzigeorgiou A. G. (2016). Diana-tarbase and diana suite tools: studying experimentally supported microrna targets. *Curr. Protoc. Bioinformatics* 55 12–14. 10.1002/cpbi.12 27603020

[B42] Peng X. Zhou D. Wang X. Hu Z. Yan Y. Huang J. (2016). Matrine suppresses proliferation and invasion of sgc7901 cells through inactivation of pi3k/akt/upa pathway. *Ann. Clin. Lab. Sci.* 46 457–462. 27650610

[B43] Plummer M. Franceschi S. Vignat J. Forman D. de Martel C. (2015). Global burden of gastric cancer attributable to*helicobacter*pylori. *Int. J. Cancer* 136 487–490. 10.1002/ijc.28999 24889903

[B44] Qian L. Liu Y. Xu Y. Ji W. Wu Q. Liu Y. (2015). Matrine derivative wm130 inhibits hepatocellular carcinoma by suppressing egfr/erk/mmp-2 and pten/akt signaling pathways. *Cancer Lett.* 368 126–134. 10.1016/j.canlet.2015.07.035 26259512

[B45] Ritchie M. E. Phipson B. Wu D. Hu Y. Law C. W. Shi W. (2015). Limma powers differential expression analyses for rna-sequencing and microarray studies. *Nucleic Acids Res.* 43:e47. 10.1093/nar/gkv007 25605792 PMC4402510

[B46] Robertson F. M. Simeone A. M. Lucci A. McMurray J. S. Ghosh S. Cristofanilli M. (2010). Differential regulation of the aggressive phenotype of inflammatory breast cancer cells by prostanoid receptors ep3 and ep4. *Cancer Am. Cancer Soc.* 116 2806–2814. 10.1002/cncr.25167 20503412 PMC2889924

[B47] Robinson M. D. McCarthy D. J. Smyth G. K. (2010). Edger: a bioconductor package for differential expression analysis of digital gene expression data. *Bioinformatics* 26 139–140. 10.1093/bioinformatics/btp616 19910308 PMC2796818

[B48] Ru J. Li P. Wang J. Zhou W. Li B. Huang C. (2014). Tcmsp: a database of systems pharmacology for drug discovery from herbal medicines. *J. Cheminform.* 6:13. 10.1186/1758-2946-6-13 24735618 PMC4001360

[B49] Rupaimoole R. Slack F. J. (2017). Microrna therapeutics: towards a new era for the management of cancer and other diseases. *Nat. Rev. Drug Discov.* 16 203–222. 10.1038/nrd.2016.246 28209991

[B50] Schmitt A. M. Chang H. Y. (2016). Long noncoding rnas in cancer pathways. *Cancer Cell.* 29 452–463. 10.1016/j.ccell.2016.03.010 27070700 PMC4831138

[B51] Schwab A. Siddiqui A. Vazakidou M. E. Napoli F. Bottcher M. Menchicchi B. (2018). Polyol pathway links glucose metabolism to the aggressiveness of cancer cells. *Cancer Res.* 78 1604–1618. 10.1158/0008-5472.CAN-17-2834 29343522

[B52] Sui H. Fu X. Pan S. Shi X. Jin B. Zhu H. (2014). PI3K/Akt/NF-κB regulate ABCB1/P-glycoprotein–mediated multidrug resistance in colon carcinoma cells. *China Oncol.* 24 106–111.

[B53] Sun M. Cao H. Sun L. Dong S. Bian Y. Han J. (2012). Antitumor activities of kushen: literature review. *Evid. Based Compl. Alt.* 2012 1–11. 10.1155/2012/373219 22969826 PMC3434675

[B54] Sung H. Ferlay J. Siegel R. L. Laversanne M. Soerjomataram I. Jemal A. (2021). Global cancer statistics 2020: globocan estimates of incidence and mortality worldwide for 36 cancers in 185 countries. *CA Cancer J. Clin.* 71 209–249. 10.3322/caac.21660 33538338

[B55] Szklarczyk D. Morris J. H. Cook H. Kuhn M. Wyder S. Simonovic M. (2017). The string database in 2017: quality-controlled protein-protein association networks, made broadly accessible. *Nucleic Acids Res.* 45 D362–D368. 10.1093/nar/gkw937 27924014 PMC5210637

[B56] Szklarczyk D. Santos A. von Mering C. Jensen L. J. Bork P. Kuhn M. (2016). Stitch 5: augmenting protein-chemical interaction networks with tissue and affinity data. *Nucleic Acids Res.* 44 D380–D384. 10.1093/nar/gkv1277 26590256 PMC4702904

[B57] Torre L. A. Bray F. Siegel R. L. Ferlay J. Lortet-Tieulent J. Jemal A. (2015). Global cancer statistics, 2012. *CA Cancer J. Clin.* 65 87–108. 10.3322/caac.21262 25651787

[B58] Trott O. Olson A. J. (2010). Autodock vina: improving the speed and accuracy of docking with a new scoring function, efficient optimization, and multithreading. *J. Comput. Chem.* 31 455–461. 10.1002/jcc.21334 19499576 PMC3041641

[B59] Tu H. Lei B. Meng S. Liu H. Wei Y. He A. (2016). Efficacy of compound kushen injection in combination with induction chemotherapy for treating adult patients newly diagnosed with acute leukemia. *Evid. Based Complement Alternat. Med.* 2016:3121402. 10.1155/2016/3121402 27738441 PMC5050378

[B60] Verdecchia A. Francisci S. Brenner H. Gatta G. Micheli A. Mangone L. (2007). Recent cancer survival in europe: a 2000–02 period analysis of eurocare-4 data. *Lancet Oncol.* 8 784–796. 17714993 10.1016/S1470-2045(07)70246-2

[B61] Vlachos I. S. Hatzigeorgiou A. G. (2017). Functional analysis of mirnas using the diana tools online suite. *Methods Mol. Biol.* 1517 25–50. 10.1007/978-1-4939-6563-2_2 27924472

[B62] Waldum H. L. Sagatun L. Mjones P. (2017). Gastrin and gastric cancer. *Front. Endocrinol. (Lausanne).* 8:1. 10.3389/fendo.2017.00001 28144230 PMC5239792

[B63] Wang B. Xu J. Wang H. Chang S. Liu N. (2017). Effect and mechanism of sophoridine to suppress hepatocellular carcinoma in vitro and vivo. *Biomed. Pharmacother.* 95 324–330. 10.1016/j.biopha.2017.08.029 28858730

[B64] Wang Q. Du H. Geng G. Zhou H. Xu M. Cao H. (2014). Matrine inhibits proliferation and induces apoptosis via bid-mediated mitochondrial pathway in esophageal cancer cells. *Mol. Biol. Rep.* 41 3009–3020. 10.1007/s11033-014-3160-3 24510386

[B65] Wang W. You R. Qin W. Hai L. Fang M. Huang G. (2015). Anti-tumor activities of active ingredients in compound kushen injection. *Acta Pharmacol. Sin.* 36 676–679. 10.1038/aps.2015.24 25982630 PMC4594177

[B66] Wu X. Li X. Fu Q. Cao Q. Chen X. Wang M. (2017). Akr1b1 promotes basal-like breast cancer progression by a positive feedback loop that activates the emt program. *J. Exp. Med.* 214 1065–1079. 10.1084/jem.20160903 28270406 PMC5379972

[B67] Xiao M. B. Jin D. D. Jiao Y. J. Ni W. K. Liu J. X. Qu L. S. (2018). Beta2-ar regulates the expression of akr1b1 in human pancreatic cancer cells and promotes their proliferation via the erk1/2 pathway. *Mol. Biol. Rep.* 45 1863–1871. 10.1007/s11033-018-4332-3 30306507

[B68] Xie S. B. He X. X. Yao S. K. (2015). Matrine-induced autophagy regulated by p53 through amp-activated protein kinase in human hepatoma cells. *Int. J. Oncol.* 47 517–526. 10.3892/ijo.2015.3023 26034977

[B69] Xu W. Lin H. Zhang Y. Chen X. Hua B. Hou W. (2011). Compound kushen injection suppresses human breast cancer stem-like cells by down-regulating the canonical wnt/β-catenin pathway. *J. Exp. Clin. Canc. Res.* 30:103. 10.1186/1756-9966-30-103 22032476 PMC3219673

[B70] Yang Y. Sun M. Yao W. Wang F. Li X. Wang W. (2020). Compound kushen injection relieves tumor-associated macrophage-mediated immunosuppression through tnfr1 and sensitizes hepatocellular carcinoma to sorafenib. *J. Immunother. Cancer* 8:e000317. 10.1136/jitc-2019-000317 32179631 PMC7073790

[B71] Yip A. M. Horvath S. (2007). Gene network interconnectedness and the generalized topological overlap measure. *BMC Bioinformatics.* 8:22. 10.1186/1471-2105-8-22 PMC1797055 17250769

[B72] Zhang D. Wu J. Wang K. Duan X. Liu S. Zhang B. (2018). Which are the best chinese herbal injections combined with xelox regimen for gastric cancer: A prisma-compliant network meta-analysis. *Medicine (Baltimore)* 97:e127. 10.1097/MD.0000000000010127 29561411 PMC5895335

[B73] Zhang D. Zheng J. Ni M. Wu J. Wang K. Duan X. (2017). Comparative efficacy and safety of chinese herbal injections combined with the folfox regimen for treating gastric cancer in china: a network meta-analysis. *Oncotarget* 8 68873–68889. 10.18632/oncotarget.20320 28978164 PMC5620304

[B74] Zhang L. Kang W. Lu X. Ma S. Dong L. Zou B. (2019). Weighted gene co-expression network analysis and connectivity map identifies lovastatin as a treatment option of gastric cancer by inhibiting hdac2. *Gene* 681 15–25. 10.1016/j.gene.2018.09.040 30266498

[B75] Zhang S. Zhang Y. Zhuang Y. Wang J. Ye J. Zhang S. (2012). Matrine induces apoptosis in human acute myeloid leukemia cells via the mitochondrial pathway and akt inactivation. *PLoS One* 7:e46853. 10.1371/journal.pone.0046853 23056487 PMC3466205

[B76] Zhang X. Hou G. Liu A. Xu H. Guan Y. Wu Y. (2019). Matrine inhibits the development and progression of ovarian cancer by repressing cancer associated phosphorylation signaling pathways. *Cell Death Dis.* 10:770. 10.1038/s41419-019-2013-3 31601793 PMC6787190

[B77] Zhang Y. Wang M. Xu X. Liu Y. Xiao C. (2019). Matrine promotes apoptosis in sw480 colorectal cancer cells via elevating mief1-related mitochondrial division in a manner dependent on lats2-hippo pathway. *J. Cell. Physiol.* 234 22731–22741. 10.1002/jcp.28838 31119752

[B78] Zhao L. Niu H. Liu Y. Wang L. Zhang N. Zhang G. (2019). Lox inhibition downregulates mmp-2 and mmp-9 in gastric cancer tissues and cells. *J. Cancer.* 10 6481–6490. 10.7150/jca.33223 31777578 PMC6856903

[B79] Zhao Z. Fan H. Higgins T. Qi J. Haines D. Trivett A. (2014). Fufang kushen injection inhibits sarcoma growth and tumor-induced hyperalgesia via trpv1 signaling pathways. *Cancer Lett.* 355 232–241. 10.1016/j.canlet.2014.08.037 25242356 PMC4253542

[B80] Zhu L. Huang S. Li J. Chen J. Yao Y. Li L. (2020). Sophoridine inhibits lung cancer cell growth and enhances cisplatin sensitivity through activation of the p53 and hippo signaling pathways. *Gene* 742:144556. 10.1016/j.gene.2020.144556 32165304

[B81] Zhuang H. Dai X. Zhang X. Mao Z. Huang H. (2020). Sophoridine suppresses macrophage-mediated immunosuppression through tlr4/irf3 pathway and subsequently upregulates cd8(+) t cytotoxic function against gastric cancer. *Biomed. Pharmacother.* 121:109636. 10.1016/j.biopha.2019.109636 31733580

