# Supplementary figures and images for: An Advanced Systems Pharmacology Strategy Reveals AKR1B1, MMP2, PTGER3 as Key Genes in the Competing Endogenous RNA Network of Compound Kushen Injection Treating Gastric Carcinoma by Integrated Bioinformatics and Experimental Verification
Source: Front Cell Dev Biol. 2021 Sep 27;9:742421. doi: 10.3389/fcell.2021.742421 (PMC8502965; doi:10.3389/fcell.2021.742421)

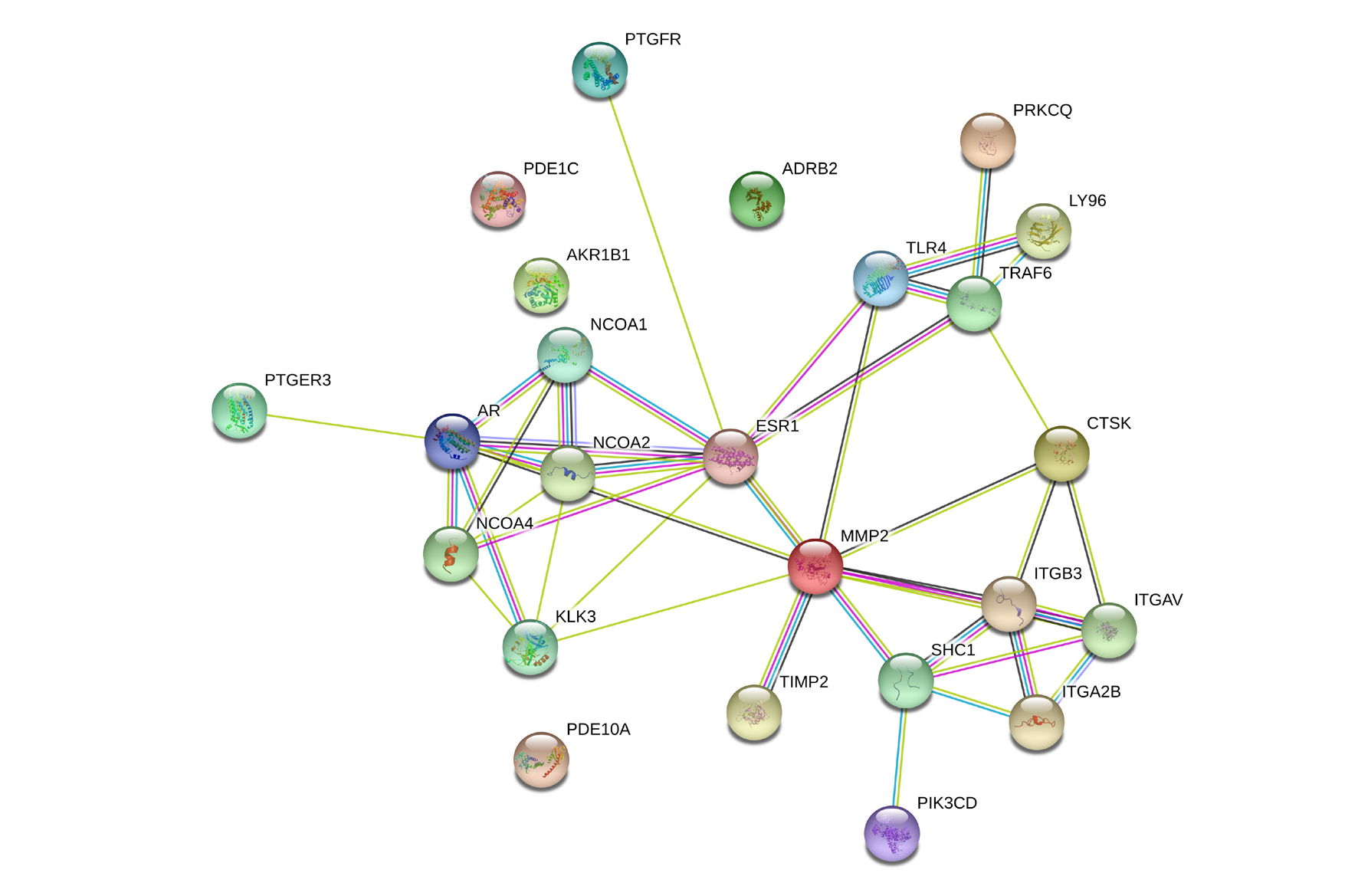

Supplement: Supplementary Figure 1 — PPI network of key genes. [file Image_1.PNG]

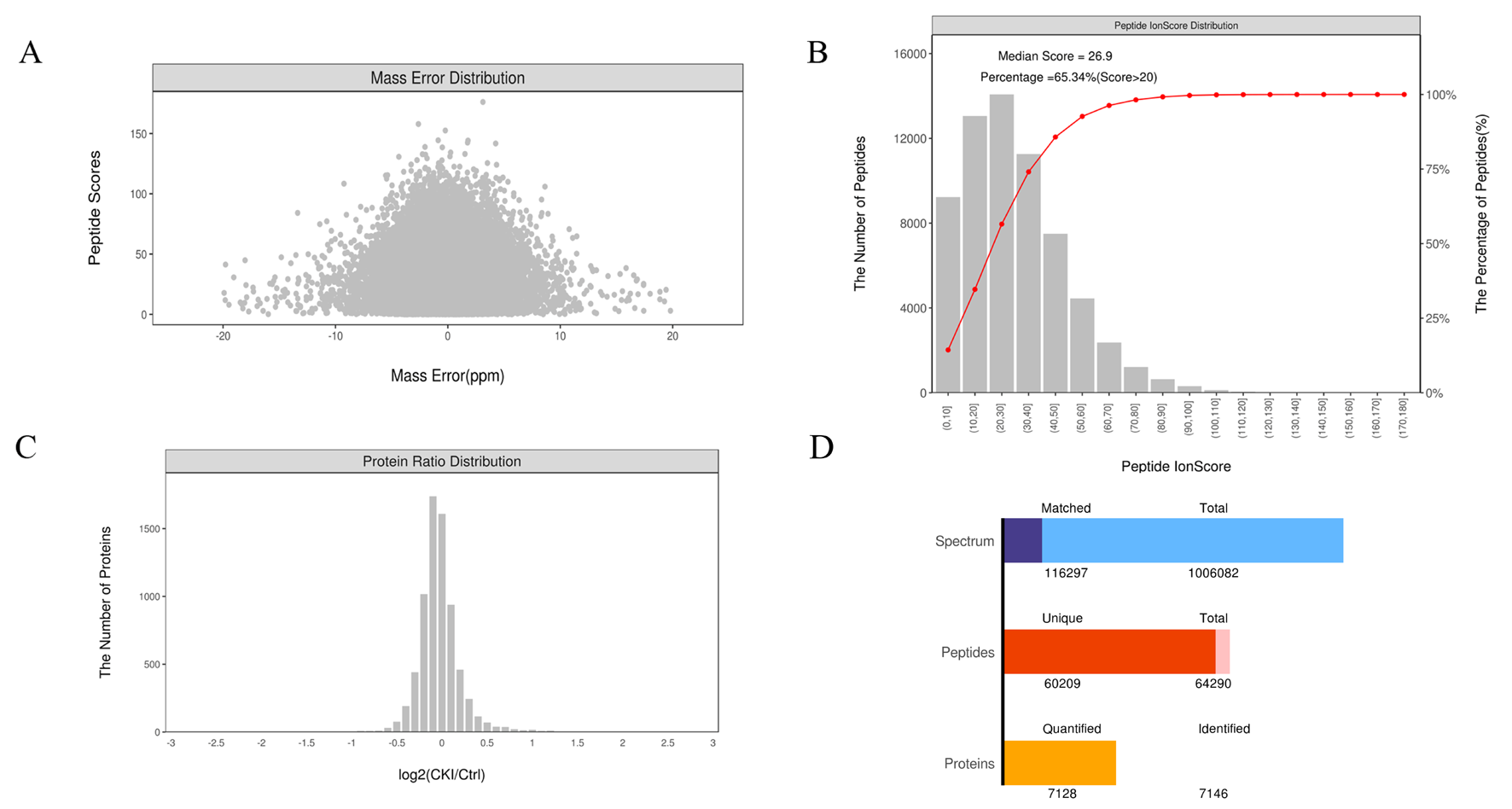

Supplement: Supplementary Figure 2 — Quality control (A–C), identification and quantification (D) of proteomics. [file Image_2.TIF]
