# Supplementary material for: An Advanced Systems Pharmacology Strategy Reveals AKR1B1, MMP2, PTGER3 as Key Genes in the Competing Endogenous RNA Network of Compound Kushen Injection Treating Gastric Carcinoma by Integrated Bioinformatics and Experimental Verification
Source: Front Cell Dev Biol. 2021 Sep 27;9:742421. doi: 10.3389/fcell.2021.742421 (PMC8502965; doi:10.3389/fcell.2021.742421)
Supplement: Supplementary Method 1 — Proteomics analysis. [file Data_Sheet_1.docx]

**Supplementary materials and methods**

**Proteomics analyses**

Protein extraction and digestion

SDT (4%SDS，100mM Tris-HCl，1mM DTT，pH7.6) buffer was used for sample lysis and protein extraction. The amount of protein was quantified with the BCA Protein Assay Kit (Bio-Rad, USA). Protein digestion by trypsin was performed according to filter-aided sample preparation (FASP) procedure described by Matthias Mann. The digest peptides of each sample were desalted on C18 Cartridges (Empore™ SPE Cartridges C18 (standard density), bed I.D. 7 mm, volume 3 ml, Sigma), concentrated by vacuum centrifugation and reconstituted in 40 µl of 0.1% (v/v) formic acid.

Filter-aided sample preparation (FASP Digestion) procedure

200 μg of proteins for each sample were incorporated into 30 μl SDT buffer (4% SDS, 100 mM DTT, 150 mM Tris-HCl pH 8.0). The detergent, DTT and other low-molecular-weight components were removed using UA buffer (8 M Urea, 150 mM Tris-HCl pH 8.0) by repeated ultrafiltration (Microcon units, 10 kD). Then 100 μl iodoacetamide (100 mM IAA in UA buffer) was added to block reduced cysteine residues and the samples were incubated for 30 min in darkness. The filters were washed with 100 μl UA buffer three times and then 100 μl 25mM NH4HCO3 buffer twice. Finally, the protein suspensions were digested with 4 μg trypsin (Promega) in 40 μl 25mM NH4HCO3 buffer overnight at 37 °C, and the resulting peptides were collected as a filtrate. The peptides of each sample were desalted on C18 Cartridges (Empore™ SPE Cartridges C18 (standard density), bed I.D. 7 mm, volume 3 ml, Sigma), concentrated by vacuum centrifugation and reconstituted in 40 µl of 0.1% (v/v) formic acid. The peptide content was estimated by UV light spectral density at 280 nm using an extinctions coefficient of 1.1 of 0.1% (g/l) solution that was calculated on the basis of the frequency of tryptophan and tyrosine in vertebrate proteins.

SDS-PAGE and labeling

20 µg of protein for each sample were mixed with 5X loading buffer respectively and boiled for 5 min. The proteins were separated on 12.5% SDS-PAGE gel (constant current 14 mA, 90 min). Protein bands were visualized by Coomassie Blue R-250 staining. 100 μg peptide mixture of each sample was labeled using TMT reagent according to the manufacturer’s instructions (Thermo Scientific).

Strong Cation Exchange (SCX) Fractionation

Labeled peptides were fractionated by SCX chromatography using the AKTA Purifier system (GE Healthcare). The dried peptide mixture was reconstituted and acidified with buffer A (10 mM KH2PO4 in 25% of ACN, pH 3.0) and loaded onto a PolySULFOETHYL 4.6 x 100 mm column (5 µm, 200 Å, PolyLC Inc, Maryland, U.S.A.). The peptides were eluted at a flow rate of 1 ml/min with a gradient of 0% buffer B (500 mM KCl, 10 mM KH2PO4 in 25% of ACN, pH 3.0) for 25 min, 0–10% buffer B during 25-32 min, 10%-20% buffer B during 32-42 min, 20-45% buffer B during 42-47 min, 45%–100% buffer B during 47-52 min, 100% buffer B during 52-60 min, and buffer B was reset to 0% after 60min. The elution was monitored by absorbance at 214 nm, and fractions were collected every 1 min. The collected fractions were desalted on C18 Cartridges (Empore™ SPE Cartridges C18 (standard density), bed I.D. 7 mm, volume 3 ml, Sigma) and concentrated by vacuum centrifugation.

LC-MS/MS analysis

LC-MS/MS analysis was performed on a Q Exactive mass spectrometer (Thermo Scientific) that was coupled to Easy nLC (Proxeon Biosystems, now Thermo Fisher Scientific) for 60/90 min. The peptides were loaded onto a reverse phase trap column (Thermo Scientific Acclaim PepMap100, 100 μm×2 cm, nanoViper C18) connected to the C18-reversed phase analytical column (Thermo Scientific Easy Column, 10 cm long, 75 μm inner diameter, 3μm resin) in buffer A (0.1% Formic acid) and separated with a linear gradient of buffer B (84% acetonitrile and 0.1% Formic acid) at a flow rate of 300 nl/min controlled by IntelliFlow technology. The mass spectrometer was operated in positive ion mode. MS data was acquired using a data-dependent top10 method dynamically choosing the most abundant precursor ions from the survey scan (300–1800 m/z) for HCD fragmentation. Automatic gain control (AGC) target was set to 3e6, and maximum inject time to 10 ms. Dynamic exclusion duration was 40.0 s. Survey scans were acquired at a resolution of 70,000 at m/z 200 and resolution for HCD spectra was set to 17,500 at m/z 200, and isolation width was 2 m/z. Normalized collision energy was 30 eV and the underfill ratio, which specifies the minimum percentage of the target value likely to be reached at maximum fill time, was defined as 0.1%. The instrument was run with peptide recognition mode enabled.

Identification and quantitation of proteins

The MS raw data for each sample were searched using the MASCOT engine (Matrix Science, London, UK; version 2.2) embedded into Proteome Discoverer 1.4 software for identification and quantitation analysis. Related parameters and instructions are as follows:

| Item | Value |
| --- | --- |
| Enzyme | Trypsin |
| Max Missed Cleavages | 2 |
| Fixed modifications | Carbamidomethyl (C),  iTRAQ 4/8plex (N-term), iTRAQ 4/8plex (K)  TMT 6/10/16 plex (N-term), TMT 6/10/16 plex (K) |
| Variable modifications | Oxidation (M) , iTRAQ 4/8plex (Y), TMT 6/10/16 plex (Y) |
| Peptide Mass Tolerance | ± 20 ppm |
| Fragment Mass Tolerance | 0.1Da |
| Database | See the project report |
| Database pattern | Decoy |
| Peptide FDR | ≤0.01 |
| Protein Quantification | The protein ratios are calculated as the median of only unique peptides of the protein |
| Experimental Bias | Normalizes all peptide ratios by the median protein ratio. The median protein ratio should be 1 after the normalization. |

Bioinformatic analysis

Cluster analysis

Cluster 3.0 (http://bonsai.hgc.jp/~mdehoon/software/cluster/software.htm) and Java Treeview software (http://jtreeview.sourceforge.net) were used to performing hierarchical clustering analysis. Euclidean distance algorithm for similarity measure and average linkage clustering algorithm (clustering uses the centroids of the observations) for clustering were selected when performing hierarchical clustering. A heat map was often presented as a visual aid in addition to the dendrogram.

Subcellular localization

CELLO (http://cello.life.nctu.edu.tw/) which is a multi-class SVM classification system, was used to predict protein subcellular localization.

Domain annotation

Protein sequences are searched using the InterProScan software to identify protein domain signatures from the InterPro member database Pfam.

GO annotation

The protein sequences of the selected differentially expressed proteins were locally searched using the NCBI BLAST+ client software (ncbi-blast-2.2.28+-win32.exe) and InterProScan to find homologue sequences, then gene ontology (GO) terms were mapped and sequences were annotated using the software program Blast2GO. The GO annotation results were plotted by R scripts.

KEGG annotation

Following annotation steps, the studied proteins were blasted against the online Kyoto Encyclopedia of Genes and Genomes (KEGG) database (http://geneontology.org/) to retrieve their KEGG orthology identifications and were subsequently mapped to pathways in KEGG.

Enrichment analysis

Enrichment analysis were applied based on the Fisher’ exact test, considering the whole quantified proteins as background dataset. Benjamini- Hochberg correction for multiple testing was further applied to adjust derived p-values. And only functional categories and pathways with p-values under a threshold of 0.05 were considered as significant.

Protein-protein interaction analysis

The protein–protein interaction (PPI) information of the studied proteins was retrieved from IntAct molecular interaction database (http://www.ebi.ac.uk/intact/) by their gene symbols or STRING software (http://string-db.org/). The results were downloaded in the XGMML format and imported into Cytoscape software (http://www.cytoscape.org/, version 3.2.1) to visualize and further analyze functional protein-protein interaction networks. Furthermore, the degree of each protein was calculated to evaluate the importance of the protein in the PPI network.
